# Glioneuronal tumor with *ATRX* alteration, kinase fusion and anaplastic features (GTAKA): a molecularly distinct brain tumor type with recurrent *NTRK* gene fusions

**DOI:** 10.1007/s00401-023-02558-0

**Published:** 2023-03-18

**Authors:** Henri Bogumil, Martin Sill, Daniel Schrimpf, Britta Ismer, Christina Blume, Ramin Rahmanzade, Felix Hinz, Asan Cherkezov, Rouzbeh Banan, Dennis Friedel, David E. Reuss, Florian Selt, Jonas Ecker, Till Milde, Kristian W. Pajtler, Jens Schittenhelm, Jürgen Hench, Stephan Frank, Henning B. Boldt, Bjarne Winther Kristensen, David Scheie, Linea C. Melchior, Viola Olesen, Astrid Sehested, Daniel R. Boué, Zied Abdullaev, Laveniya Satgunaseelan, Ina Kurth, Annekatrin Seidlitz, Christine L. White, Ho-Keung Ng, Zhi-Feng Shi, Christine Haberler, Martina Deckert, Marco Timmer, Roland Goldbrunner, Arnault Tauziède-Espariat, Pascale Varlet, Sebastian Brandner, Sanda Alexandrescu, Matija Snuderl, Kenneth Aldape, Andrey Korshunov, Olaf Witt, Christel Herold-Mende, Andreas Unterberg, Wolfgang Wick, Stefan M. Pfister, Andreas von Deimling, David T. W. Jones, Felix Sahm, Philipp Sievers

**Affiliations:** 1 Department of Neuropathology, Institute of Pathology, University Hospital Heidelberg, Heidelberg, Germany; 2 Clinical Cooperation Unit Neuropathology, German Consortium for Translational Cancer Research (DKTK), German Cancer Research Center (DKFZ), Heidelberg, Germany; 3 Hopp Children’s Cancer Center Heidelberg (KiTZ), Heidelberg, Germany; 4 Division of Pediatric Neurooncology, German Consortium for Translational Cancer Research (DKTK), German Cancer Research Center (DKFZ), Heidelberg, Germany; 5 Division of Pediatric Glioma Research, German Cancer Research Center (DKFZ), Heidelberg, Germany; 6 Faculty of Biosciences, Heidelberg University, Heidelberg, Germany; 7 Clinical Cooperation Unit Pediatric Oncology, German Consortium for Translational Cancer Research (DKTK), German Cancer Research Center (DKFZ), Heidelberg, Germany; 8 Department of Pediatric Oncology, Hematology, Immunology and Pulmonology, University Hospital Heidelberg, Heidelberg, Germany; 9 National Center for Tumor Diseases (NCT), Heidelberg, Germany; 10 Center for Neuro-Oncology, Comprehensive Cancer Center Tübingen-Stuttgart, University Hospital Tübingen, Eberhard-Karls-University Tübingen, Tübingen, Germany; 11 German Cancer Consortium (DKTK), DKFZ Partner Site Tübingen, Tübingen, Germany; 12 Department of Neuropathology, University Hospital Tübingen, Eberhard-Karls-University Tübingen, Tübingen, Germany; 13 Division of Neuropathology, Institute for Pathology, University Hospital Basel, Basel, Switzerland; 14 Department of Pathology, Odense University Hospital, Odense, Denmark; 15 Department of Clinical Research, University of Southern Denmark, Odense, Denmark; 16 Department of Pathology, The Bartholin Institute, Rigshospitalet, Copenhagen University Hospital, Copenhagen, Denmark; 17 Department of Clinical Medicine and Biotech Research & Innovation Centre (BRIC), University of Copenhagen, Copenhagen, Denmark; 18 Department of Pathology, Rigshospitalet, Copenhagen University Hospital, Copenhagen, Denmark; 19 Spine Unit, Rigshospitalet, Copenhagen University Hospital, Copenhagen, Denmark; 20 Department of Pediatrics and Adolescent Medicine, Copenhagen University Hospital, Copenhagen, Denmark; 21 Department of Pathology and Laboratory Medicine, Nationwide Children’s Hospital and the Ohio State University, Columbus, OH USA; 22 Laboratory of Pathology, Center for Cancer Research, National Cancer Institute, National Institutes of Health, Bethesda, MD USA; 23 Department of Neuropathology, Royal Prince Alfred Hospital, Sydney, NSW Australia; 24 Division of Radiooncology-Radiobiology, German Consortium for Translational Cancer Research (DKTK), German Cancer Research Center (DKFZ), Heidelberg, Germany; 25 Department of Radiation Oncology, Faculty of Medicine and University Hospital Carl Gustav Carus, Technische Universität Dresden, Dresden, Germany; 26 National Center for Tumor Diseases (NCT), Partner Site Dresden, Dresden, Germany; 27 OncoRay–National Center for Radiation Research in Oncology, Faculty of Medicine and University Hospital Carl Gustav Carus, Technische Universität Dresden, Helmholtz-Zentrum Dresden-Rossendorf, Dresden, Germany; 28 German Cancer Research Center (DKFZ), Heidelberg and German Consortium for Translational Cancer Research (DKTK) Partner Site, Dresden, Germany; 29 German Cancer Research Center (DKFZ), Heidelberg, Germany; 30 Faculty of Medicine and University Hospital Carl Gustav Carus, Technische Universität Dresden, Dresden, Germany; 31 Helmholtz Association/Helmholtz-Zentrum Dresden-Rossendorf (HZDR), Dresden, Germany; 32 Hudson Institute of Medical Research, Clayton, Australia; 33 Department of Molecular and Translational Science, Monash University, Clayton, Australia; 34 Victorian Clinical Genetics Services, Parkville, Australia; 35 Department of Anatomical and Cellular Pathology, The Chinese University of Hong Kong, Hong Kong, China; 36 Hong Kong and Shanghai Brain Consortium (HSBC), Hong Kong, China; 37 Department of Neurosurgery, Huashan Hospital, Fudan University, Shanghai, China; 38 Division of Neuropathology and Neurochemistry, Department of Neurology, Medical University of Vienna, Vienna, Austria; 39 Institute of Neuropathology, Faculty of Medicine and University Hospital Cologne, University of Cologne, Cologne, Germany; 40 Laboratory for Neurooncology and Experimental Neurosurgery, Department of General Neurosurgery, Center for Neurosurgery, Faculty of Medicine and University Hospital Cologne, University of Cologne, Cologne, Germany; 41 Department of Neuropathology, GHU Paris - Psychiatry and Neuroscience, Sainte-Anne Hospital, Paris, France; 42 Institut de Psychiatrie et Neurosciences de Paris (IPNP), UMR S1266, INSERM, IMA-BRAIN, Paris, France; 43 Division of Neuropathology, National Hospital for Neurology and Neurosurgery, University College London Hospitals NHS Foundation Trust, Queen Square, London, UK; 44 Department of Neurodegenerative Disease, UCL Queen Square Institute of Neurology, Queen Square, London, UK; 45 Department of Pathology, Boston Children’s Hospital, Boston, MA USA; 46 Department of Pathology, NYU Langone Medical Center, New York, NY USA; 47 Division of Experimental Neurosurgery, Department of Neurosurgery, University Hospital Heidelberg, Heidelberg, Germany; 48 Department of Neurosurgery, University Hospital of Heidelberg, Heidelberg, Germany; 49 Clinical Cooperation Unit Neurooncology, German Consortium for Translational Cancer Research (DKTK), German Cancer Research Center (DKFZ), Heidelberg, Germany; 50 Department of Neurology and Neurooncology Program, National Center for Tumor Diseases (NCT), Heidelberg University Hospital, Heidelberg, Germany

**Keywords:** Glioneuronal tumor, Gene fusion, *NTRK*, *ATRX*, DNA methylation

## Abstract

Glioneuronal tumors are a heterogenous group of CNS neoplasms that can be challenging to accurately diagnose. Molecular methods are highly useful in classifying these tumors—distinguishing precise classes from their histological mimics and identifying previously unrecognized types of tumors. Using an unsupervised visualization approach of DNA methylation data, we identified a novel group of tumors (*n* = 20) that formed a cluster separate from all established CNS tumor types. Molecular analyses revealed *ATRX* alterations (in 16/16 cases by DNA sequencing and/or immunohistochemistry) as well as potentially targetable gene fusions involving receptor tyrosine-kinases (RTK; mostly *NTRK1-3*) in all of these tumors (16/16; 100%). In addition, copy number profiling showed homozygous deletions of *CDKN2A/B* in 55% of cases. Histological and immunohistochemical investigations revealed glioneuronal tumors with isomorphic, round and often condensed nuclei, perinuclear clearing, high mitotic activity and microvascular proliferation. Tumors were mainly located supratentorially (84%) and occurred in patients with a median age of 19 years. Survival data were limited (*n* = 18) but point towards a more aggressive biology as compared to other glioneuronal tumors (median progression-free survival 12.5 months). Given their molecular characteristics in addition to anaplastic features, we suggest the term glioneuronal tumor with *ATRX* alteration, kinase fusion and anaplastic features (GTAKA) to describe these tumors. In summary, our findings highlight a novel type of glioneuronal tumor driven by different RTK fusions accompanied by recurrent alterations in *ATRX* and homozygous deletions of *CDKN2A/B*. Targeted approaches such as NTRK inhibition might represent a therapeutic option for patients suffering from these tumors.

**Supplementary Information:**

The online version contains supplementary material available at 10.1007/s00401-023-02558-0.

## Introduction

Glioneuronal tumors represent a histologically and molecularly heterogeneous group of rare neoplasms of the central nervous system (CNS) [4, 15]. Accurate diagnosis of these tumors is often challenging due to overlapping morphological and immunohistochemical features. The recently published fifth edition of the World Health Organization (WHO) classification of CNS tumors comprises 15 different types of glioneuronal and neuronal tumors, which correspond most frequently to WHO grade 1 or 2 [15].

In recent years, CNS tumor classification has been greatly influenced by novel technologies such as DNA methylation analysis and led to the identification of numerous previously unrecognized, relatively rare types of brain tumors, some of them with a wide range of histopathological appearances [1, 5, 6, 19, 20, 24]. Furthermore, comprehensive molecular profiling of glioneuronal tumors revealed several ‘druggable’ targets mainly affecting the mitogen-activated protein kinase (MAPK) pathway, including *BRAF* as well as *FGFR* and *NTRK* gene family alterations as found e.g. in ganglioglioma [18], dysembryoplastic neuroepithelial tumor [17], rosette-forming glioneuronal tumor [9, 16, 23], diffuse leptomeningeal glioneuronal tumor [8], extraventricular neurocytoma [16, 26], polymorphous low-grade neuroepithelial tumors of the young (PLNTY) [11], or the recently described group of glioneuronal tumors driven by different kinase-fusions [25, 28].

Here, we describe a molecularly distinct type of glioneuronal tumors using a DNA methylation-based approach in combination with targeted next-generation DNA and RNA sequencing. In addition to molecular characterization, tumors were investigated through an extensive histomorphological and immunohistochemical workup and through retrospective analysis of the available clinical data.

## Materials and methods

### Collection of tissue samples and clinical data

Case selection of this series was based on unsupervised visualization of genome-wide DNA methylation data (more than 100,000 CNS tumor samples) that revealed an epigenetically distinct cluster of tumors (*n* = 20). Tumor samples and retrospective clinical data were obtained from multiple international institutes, with a subset of cases initially uploaded to the www.molecularneuropathology.org platform. Collection and analysis of tissue samples and clinical data was performed in accordance with local ethics regulations (ethical vote S-318/2022).

### Histology and immunohistochemistry

Histological and immunohistochemical review was performed on a subset of tumor samples with available material for extensive histopathological workup (*n* = 18). Hematoxylin–eosin (H&E) and reticulin staining was performed according to standard protocols. Immunohistochemistry was carried out on a Ventana BenchMark ULTRA Immunostainer (Ventana Medical Systems, Tucson, AZ, USA). Following Antibodies were used: ATRX (mouse monoclonal, clone BSB-108, dilution 1:2000, Bio SB, Santa Barbara, CA, USA), CD34 (mouse monoclonal, clone QBEnd/10, undiluted, Roche Ventana, Basel, Switzerland), Class III ß-tubulin (mouse monoclonal, clone TU-20, dilution 1:100, Abcam, Cambridge, UK), GFAP (mouse monoclonal, clone GA5, dilution 1:2000, Cell Signaling, Danvers, MA, USA), Ki-67 (mouse monoclonal, clone MIB-1, dilution 1:100, Dako Agilent, Santa Clara, CA, USA), MAP2 (mouse monoclonal, clone HM-2, dilution 1:15,000, Sigma-Aldrich, St. Louis, MO, USA), Neu-N (mouse monoclonal, clone A60, dilution 1:100, Merck, Darmstadt, Germany), NSE (mouse monoclonal, clone MIG-N3, dilution 1:4, Linaris, Dossenheim, Germany), Olig2 (rabbit monoclonal, clone EPR2673, dilution 1:50, Abcam), Synaptophysin (rabbit monoclonal,clone MRQ-40, dilution 1:50, Merck). Class III ß-tubulin, Neu-N, NSE, Olig2, MAP2, Synaptophysin and GFAP immunohistochemistry was performed with the Ventana UltraView DAB IHC Detection Kit. For ATRX, CD34 and Ki-67 the Ventana OptiView DAB IHC Detection Kit (Ventana Medical Systems) were used. Slides were scanned with an Aperio AT2 scanner and visualized with Aperio Image Scope v12.4.3.7001 (Aperio, Leica Biosystems, Deer Park, IL, USA). For one case available material only allowed H&E staining while immunohistochemical information was submitted via the cooperating center. For six cases histological and immunohistochemical review was performed in collaborating institutes and information or scans were digitally evaluated.

### DNA methylation profiling and copy number analysis

DNA methylation analysis was performed using formalin-fixed paraffin-embedded (FFPE) or fresh-frozen tissue samples. Raw data was generated at the Department of Neuropathology Heidelberg, the German Cancer Research Center (DKFZ) or at respective collaborating institutes using the Infinium MethylationEPIC (850k) or Infinium HumanMethylation450 (450k) BeadChip array (Illumina, San Diego, CA, USA) according to the manufacturer’s instructions and as previously described [5]. All computational analyses were performed using R version 4.6.1 (R Development Core Team 2020, https://www.R-project.org). *O6-methylguanine-DNA methyltransferase* (MGMT) -promoter methylation status was evaluated using the method described by Bady et al. [3]. For an unsupervised hierarchical cluster analysis of tumors and reference samples, the 10,000 most variably methylated CpG sites across the dataset according to median absolute deviation were selected. Clustering was done using the Euclidean distance and Ward linkage after adjustment for FFPE versus frozen material. To perform unsupervised non-linear dimension reduction, the remaining probes after standard filtering were used to calculate the 1-variance weighted Pearson correlation between samples. The resulting distance matrix was used as input for t-SNE analysis (t-distributed stochastic neighbor embedding). The following non-default parameters were applied: is distance = T, theta = 0, pca = F, max_iter = 10,000 perplexity = 30.

### RNA sequencing and fusion calling

RNA sequencing for the purpose of gene fusion calling was performed in 16/20 (80%) of the cases. For 14 cases RNA sequencing was performed on a NextSeq 500 or NovaSeq 6000 instrument (Illumina) at the Department of Neuropathology Heidelberg as previously described [27]. In addition, one case (#14) was analyzed at the Department of Pathology Copenhagen (Rigshospitalet, Copenhagen University Hospital, Denmark) using an Archer FusionPlex Solid tumor panel (Invitae, Boulder, CO, USA) on an Ion Torrent S5 Prime platform (Thermo Fisher, Waltham, MA, USA). One case (#2) was analyzed at the Department of Pathology at Boston Children’s Hospital and Harvard Medical School (Boston, MA, USA) as previously described [29].

### Targeted next-generation DNA sequencing and mutational analysis

DNA sequencing were performed in all cases with available material for DNA extraction (15/20 cases, 75%). The majority of cases (12/14, 86%) were sequenced at the Department of Neuropathology Heidelberg using a capture-based next-generation DNA sequencing approach on a NextSeq 500 or NovaSeq 6000 instrument (Illumina) applying a custom brain tumor panel as previously described [21]. For one case (#14) mutational analysis was performed at the Department of Pathology Copenhagen (Rigshospitalet, Copenhagen University Hospital, Denmark) using an AmpliSeq NGS neuropanel on an Ion Torrent S5 Prime platform (Thermo Fisher). Additionally, one case (#2) was sequenced at the Department of Pathology, Boston Children’s Hospital and Harvard Medical School (Boston, MA, USA) as previously described [29].

### Statistical analysis

Survival analysis was performed using GraphPad Prism 9 (GraphPad Software, La Jolla, CA, USA). Data on survival could be retrospectively retrieved for 18 of 20 (90%) patients. Overall survival (OS) and progression-free survival (PFS) probabilities were displayed using Kaplan–Meier method.

## Results

### Epigenetic profiling reveals a molecularly distinct group of neuroepithelial tumors

**Fig.1:**
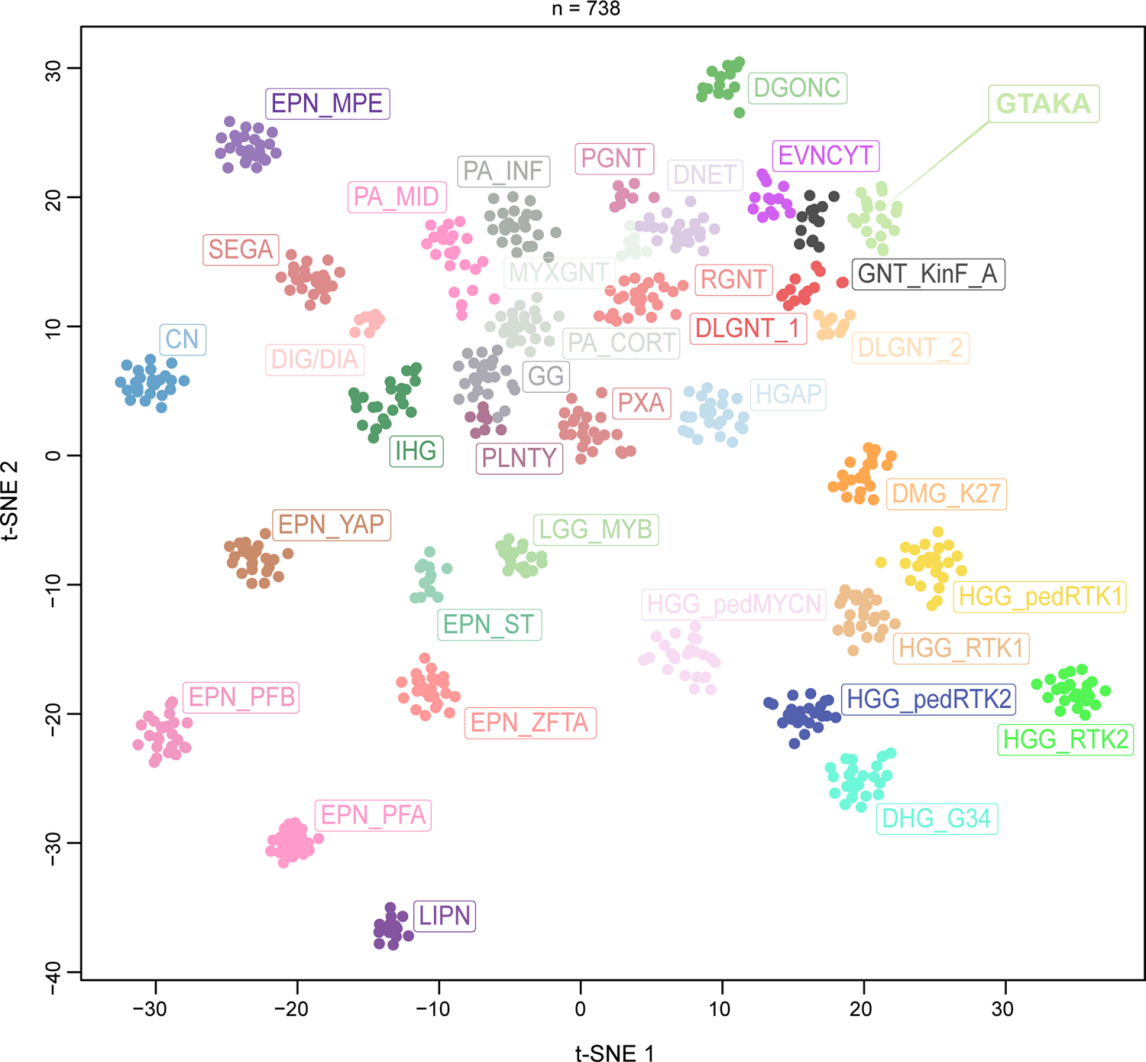
Unsupervised, non-linear t-distributed stochastic neighbor embedding (t-SNE) projection of DNA methylation array profiles from 738 tumor samples. DNA methylation profiling reveals a molecular distinct group of glioneuronal tumors (GTAKA). Reference DNA methylation classes: dysembryoplastic neuroepithelial tumor (DNET), rosette-forming glioneuronal tumor (RGNT), diffuse leptomeningeal glioneuronal tumor subtype 1 (DLGNT_1), diffuse leptomeningeal glioneuronal tumor subtype 2 (DLGNT_2), extraventricular neurocytoma (EVNCYT), papillary glioneuronal tumor (PGNT), ganglioglioma (GG), polymorphous low-grade neuroepithelial tumor of the young (PLNTY), myxoid glioneuronal tumor, PDGFRA-mutant (MYXGNT), diffuse glioneuronal tumor with oligodendroglioma-like features and nuclear clusters (DGONC), glioneuronal tumor kinase-fused (GNT_KinF_A), central neurocytoma (CN), cerebellar liponeurocytoma (LIPN), desmoplastic infantile ganglioglioma / desmoplastic infantile astrocytoma (DIG/DIA), angiocentric glioma MYB/MYBL1-altered (LGG_MYB), pilocytic astrocytoma hemispheric (PA_CORT), pilocytic astrocytoma infratentorial (PA_INF), pilocytic astrocytoma midline (PA_MID), pleomorphic xanthoastrocytoma (PXA), infant-type hemispheric glioma (IHG), high-grade astrocytoma with piloid features (HGAP), subependymal giant cell astrocytoma (SEGA), diffuse midline glioma, H3 K27-altered (DMG_K27), diffuse hemispheric glioma, H3 G34-mutant (DHG_G34), glioblastoma, IDH-wildtype, RTK1 subtype (HGG_RTK1), glioblastoma, IDH-wildtype, RTK2 subtype (HGG_RTK2), diffuse pediatric-type high-grade glioma, RTK1 subtype (HGG_pedRTK1), diffuse pediatric-type high-grade glioma, RTK2 subtype (HGG_pedRTK2) and diffuse pediatric-type high-grade glioma, MYCN subtype (HGG_pedMYCN), supratentorial ependymoma, ZFTA-fused (EPN_ZFTA), supratentorial ependymoma, YAP1-fused (EPN_YAP), supratentorial ependymoma, NOS (EPN_ST), posterior fossa group A ependymoma (EPN_PFA), posterior fossa group B ependymoma (EPN_PFB), myxopapillary enpendymoma (EPN_MPE)

By unsupervised visualization of genome-wide DNA methylation data (t-SNE analysis) from more than 100,000 CNS tumor samples, we identified a distinct group of tumors (*n* = 20) that formed a cluster separate from all established DNA methylation classes. All of these cases occurred in the CNS and originally received various different histological diagnoses as further described below. A focused t-distributed stochastic neighbor embedding (t-SNE) analysis using a reference cohort of 718 neuroepithelial tumors including different glial and glioneuronal tumors confirmed the distinct clustering (Fig. 1). No similarity was seen with the recently described tumor type ‘glioneuronal tumor kinase-fused A’ (Supplementary Fig. 1) [25].

**Fig.2:**
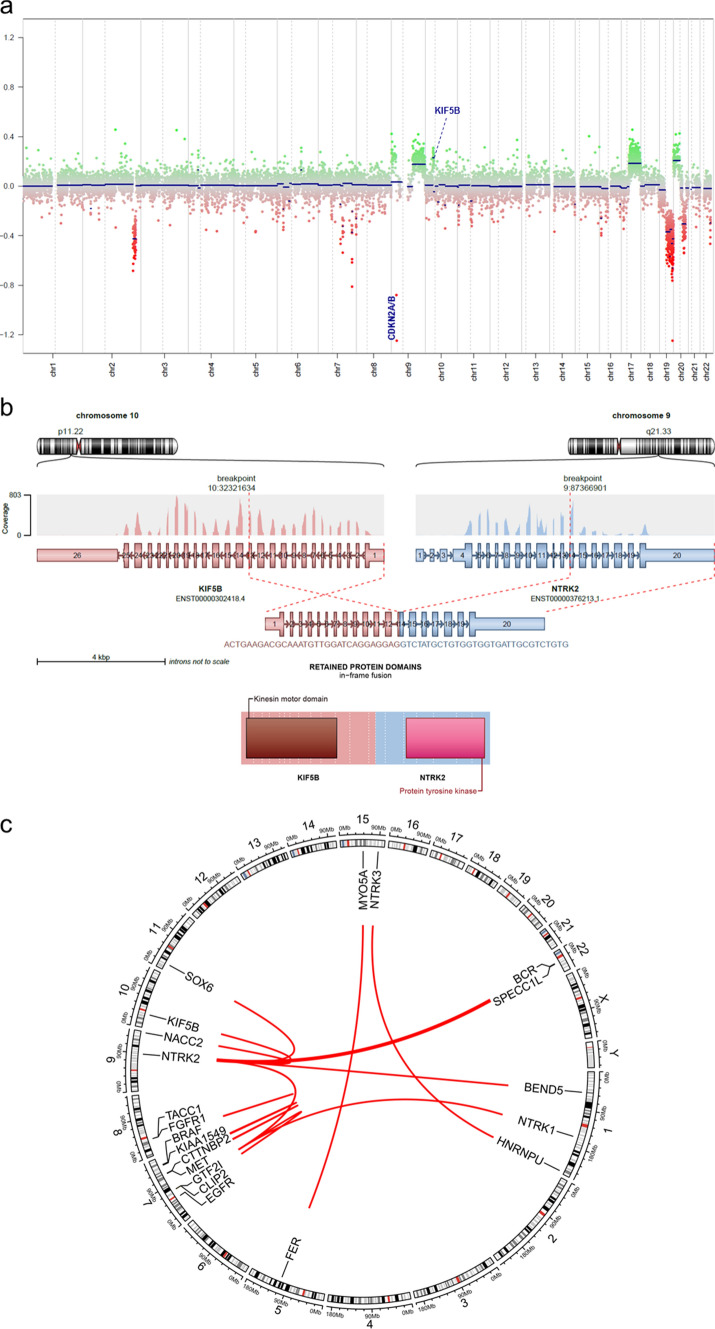
Copy-number profile derived from DNA methylation array data showing a homozygous deletion of *CDKN2A/B* as well as structural alterations affecting chromosome 9q and 10p around the *NTRK2* and *KIF5B* loci (**a**). Visualization of the *KIF5B::NTRK2* gene fusion confirmed by RNA sequencing (**b**). Circos plot of the different gene fusions detected in the series (lines link fusion gene partners according to chromosomal location; **c**)

Analysis of copy number variations derived from the DNA methylation data showed a homozygous deletion at chr9p21 including *CDKN2A/B* in 11 of 20 (55%) cases (Table 1 and Fig. 2a). In addition, a heterozygous deletion of *CDKN2A/B* was observed in one of the cases (Table 1 and Supplementary Table 1, online resource). Further recurrent copy number alterations included: segmental or whole gains of chr9q in 12/20 (60%) and chr17q in 12/20 (60%) of the cases, as well as a segmental or whole loss of chr19q in 11/20 (55%) cases. In addition, several tumors (7 out of 20) showed indication for a gene fusion based on their copy number profile (Fig. 2a and Supplementary Table 1, online resource). This included three *NTRK* fusions with focal gain or loss located at the gene locus of the respective fusion partner (Fig. 2b) as well as one case with a *KIAA1549::BRAF* fusion, two cases with a *FGFR1::TACC1* fusion and one case with *CLIP2::EGFR* fusion (Supplementary Table. 1, online resource). All recurrent copy number variations detected are given in a summary chart in the supplement (Supplementary Table 1 and Supplementary Figs. 2, 3, 4, 5, 6, 7, 8, 9, 10, 11, 12, 13, 14, 15, 16, 17, 18, online resource).

**Table 1:** Clinico-pathological characteristics and key molecular finding of the series

#	Sex	Age (years)	Tumor location	Gene fusion	CDKN2A/B status	ATRX alteration*
01	F	16	Supratentorial, frontal, left	*SPECC1L::NTRK2*	Balanced	Yes
02	F	16	Supratentorial, frontal, left	*SPECC1L::NTRK2*	Homozygous loss	Yes
03	M	8	Brain NOS	*BEND5::NTRK2*	Balanced	Yes
04	F	33	Infratentorial, posterior fossa	*NACC2::NTRK2*	Homozygous loss	Yes
05	M	42	Supratentorial, third ventricle	*SOX6::NTRK2*	Heterozygous loss	Yes
06	F	32	Infratentorial, cerebellar	*KIF5B::NTRK2*	Homozygous loss	Yes
07	M	14	Supratentorial, parieto-occipital, right	*CLIP2::NTRK2*	Balanced	Yes
08	F	56	Supratentorial, frontal, right	*BCR::NTRK2*	Homozygous loss	Yes
09	F	18	Supratentorial, frontal, left	*GTF2I::NTRK2*	Homozygous loss	Yes
10	M	65	Supratentorial, frontal, right	*GTF2I::NTRK1*	Balanced	Yes
11	M	4	Supratentorial, hemispheric	*HNRNPU::NTRK3*	Homozygous loss	Yes
12	F	20	Supratentorial	*FGFR1::TACC1*	Balanced	NA
13	M	23	Supratentorial, frontal, right	*CLIP2::EGFR*	Balanced	Yes
14	M	76	Spinal, th7-th9, intramedullary	*KIAA1549::BRAF*	Balanced	Yes
15	F	15	Supratentorial, lateral ventricle, right	*MYO5A::FER*	Homozygous loss	Yes
16	F	13	Supratentorial, temporal, left	*CTTNBP2::MET*	Homozygous loss	Yes
17	M	12	Supratentorial, frontal, right, disseminated	NA	Homozygous loss	NA
18	F	31	Supratentorial, thalamic	NA	Homozygous loss	Yes
19	F	76	Supratentorial, occipital, right	NA	Homozygous loss	NA
20	F	6	Supratentorial	NA	Balanced	NA

^*^ATRX loss detected by immunohistochemistry and/or ATRX mutation detected by NGS

### RNA sequencing identifies oncogenic gene fusions involving different receptor tyrosine-kinases as a frequent event in this novel group of neuroepithelial tumors

RNA sequencing for the purpose of gene fusion detection could be performed in 16 of 20 cases and revealed rearrangements involving different receptor tyrosine-kinases (RTKs) in all samples (Table 1 and Fig. 2c). Eleven of the cases harbored rearrangements involving the *NTRK* gene family, with the most common fusion partner being *NTRK2* (*n* = 9). In addition, one *NTRK1* and one *NTRK3* fusion was detected in this series (Supplementary Table 1, online resource). In all fusions NTRK1-3 served as the 3′ partner and the tyrosine kinase domain was conserved as a part of the fusion product (Fig. 2b). *NTRK* fusion partners were highly variable and included: *SPECC1L*, *BEND5*, *NACC2*, *SOX6*, *KIF5B*, *CLIP2*, *BCR*, *GTF2I* and *HNRNPU* (Fig. 2c). Further oncogenic rearrangements detected by RNA sequencing included an *FGFR1::TACC1*, *CLIP2::EGFR*, *KIAA1549::BRAF*, *MYO5A::FER* and *CTTNBP2::MET* fusion (Fig. 2c). Detailed information about fusion partners, breakpoints and indication of the gene fusions based on the copy number variations are given in the supplement (Supplementary Table 1 and Supplementary Figs. 2, 3, 4, 5, 6, 7, 8, 9, 10, 11, 12, 13, 14, 15, 16, 17, 18, online resource).

### Mutational analysis identifies recurrent alterations in *ATRX* accompanied by an immunohistochemical loss

**Fig.3:**
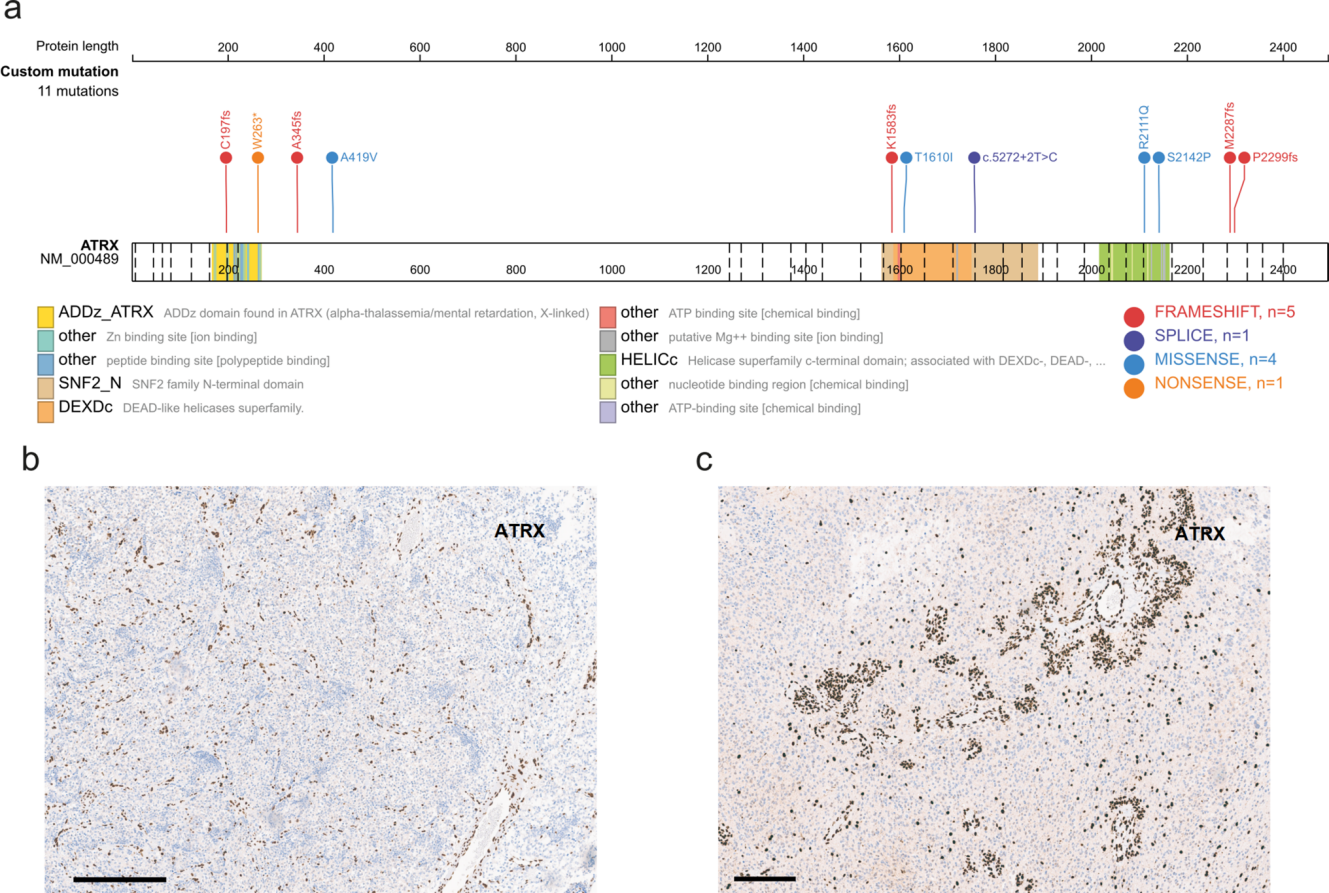
Visualization of the *ATRX* mutation profile in the investigated cohort was created using the online tool ProteinPaint available at https://proteinpaint.stjude.org/ (**a**). Immunohistochemical loss of ATRX expression in the tumor cells of case #01 and #18 (**b**, **c**). Scale bars 200 µm

Targeted next-generation DNA sequencing was performed in 15 of 20 cases (75%) and revealed recurrent alterations in *ATRX* in 12 of 15 cases (80%). The mutational spectrum includes different frameshift, missense and nonsense mutations (Fig. 3a). In three of the cases an *ATRX* alteration was not detected by sequencing, however, an immunohistochemical loss of nuclear ATRX expression was present in tumor cells (Fig. 3b, c). In two cases nuclear ATRX expression was retained, but inactivating *ATRX* mutations were detected. All detected ATRX alterations as well as the immunohistochemical status of the single cases are also provided in Supplementary Table 1 (online resource). No additional recurrent alteration was detected by DNA sequencing.

### Histological and immunohistochemical review reveals glioneuronal tumors with clear cell morphology, condensed nuclei and anaplastic features

**Fig. 4:**
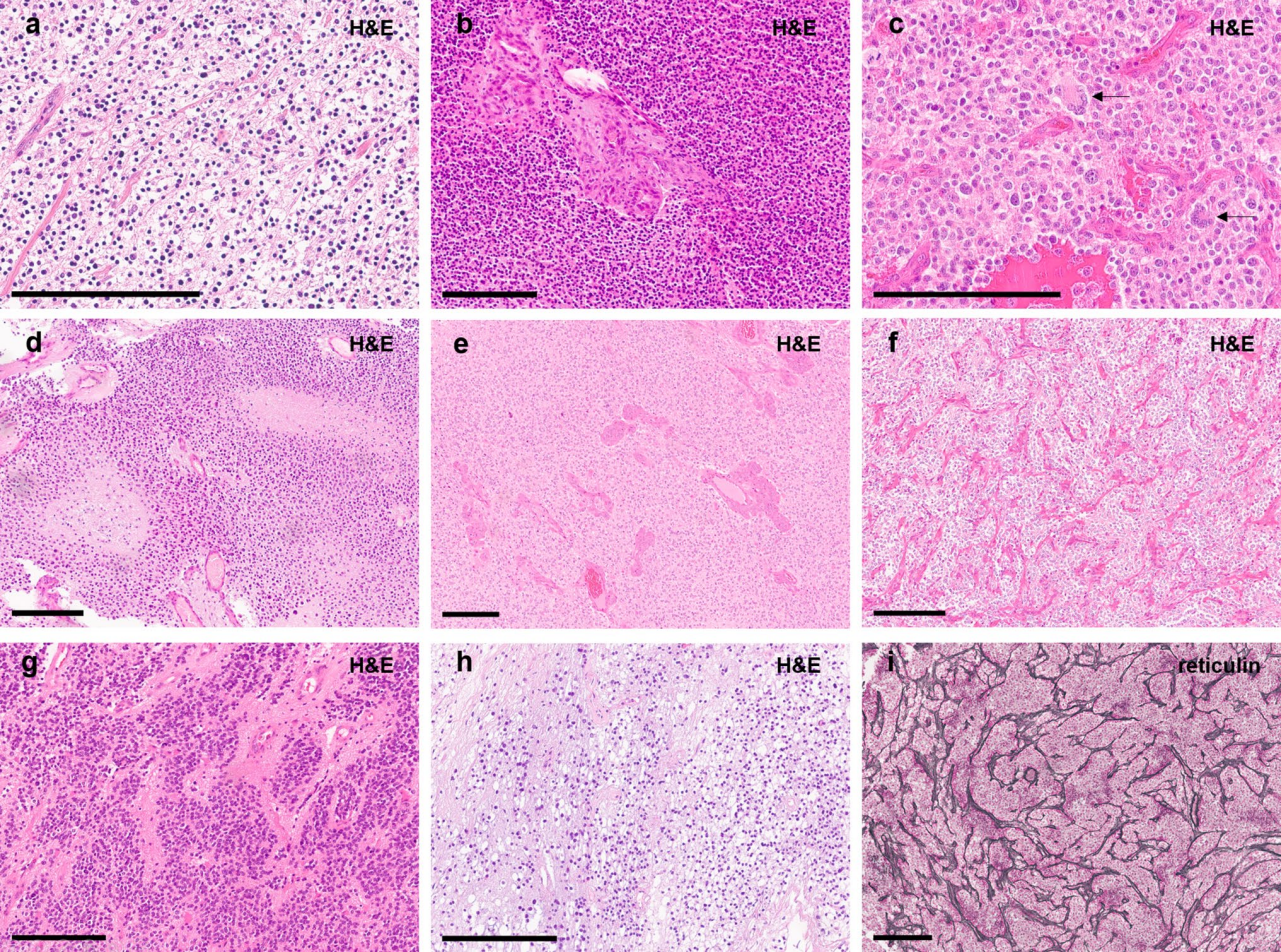
Typical histological features of tumors within this series showing tumor cells with inconspicuous nuclei and perinuclear clearing (**a**, **b**). Some cases show multinucleated cells (**c**, indicated by arrows). Cell density is variable (**b**, **h**). Tumors are densely vascularized (note the typical pattern, as f and i show two different cases). Microvascular proliferation is frequently observed (**b**, **e**). Necrosis is present in a subset of cases (**d**). An angiocentric arrangement was seen in single cases (**g**). Scale bars 200 µm

**Fig. 5:**
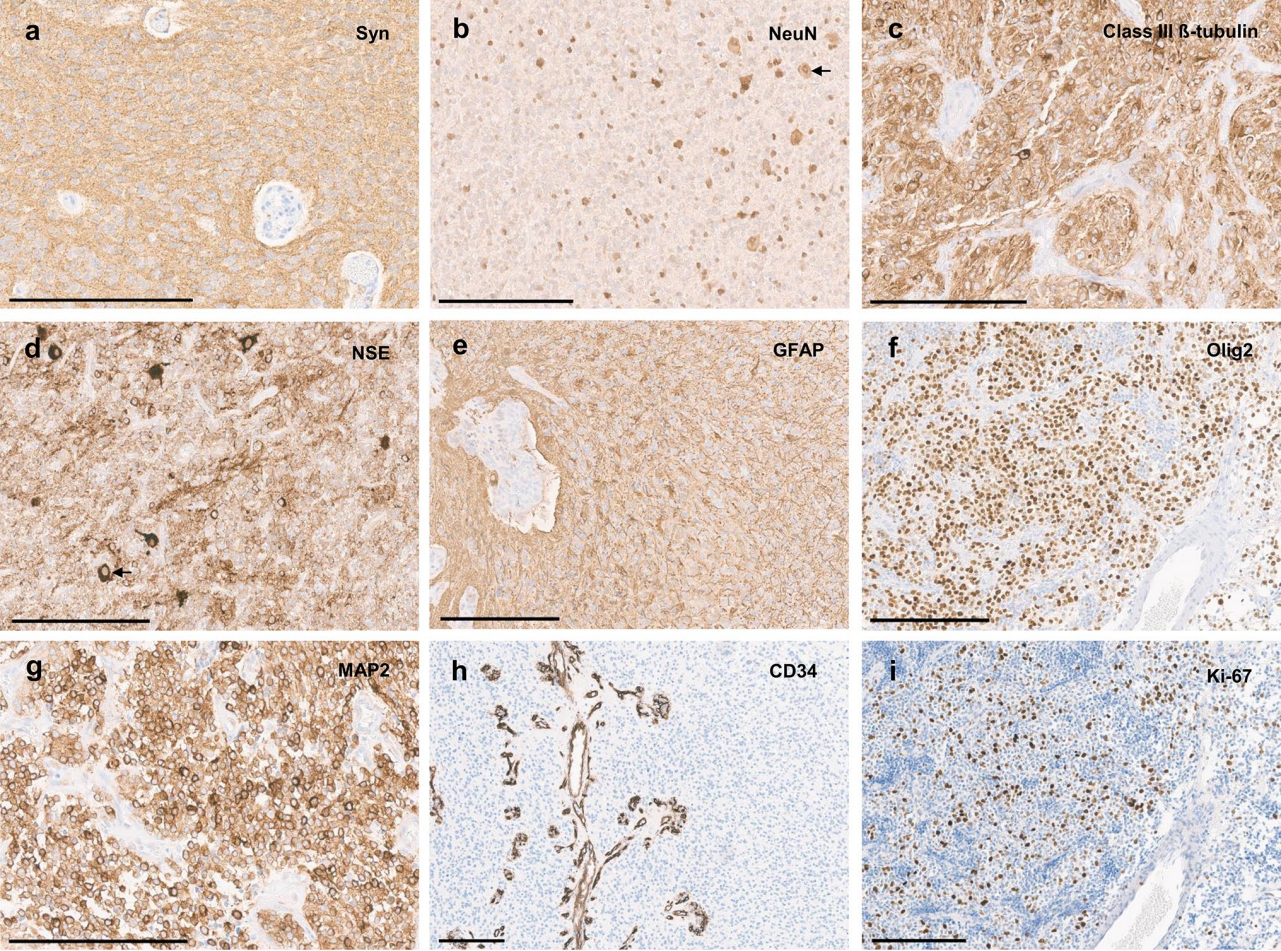
Immunohistochemistry reveals diffuse positivity for synaptophysin with a weak or moderate cytoplasmic reactivity, while the tumor cell matrix shows a consistently strong staining (**a**). The majority of tumor cells shows negative or weak NeuN staining, although scattered positive cells are frequently present (**b**). Class III ß-tubulin (**c**) and NSE (**d**) are positive in the tumors, although sometimes restricted to a proportion of neoplastic cells. GFAP staining shows strong positivity of the tumor cell matrix with variable cytoplasmic reactivity between the cases and/or different areas within the same tumor (**e**). Olig2 staining is usually strongly positive (**f**). MAP2 staining is positive in all tumors (**g**). CD34 expression is restricted to the vessels (**h**). Ki-67 labeling index varies between the different cases, here showing a moderate proliferative activity (**f**). Scale bars 200 µm

Histologically, tumor cells typically showed perinuclear clearing (14/16, 87%), at first sight mimicking oligodendroglioma or neurocytoma (Fig. 4a). Nuclei were mostly isomorphous and round, often with remarkably condensed chromatin (Fig. 4a, b). Multinucleated cells were observed frequently (11/15, 73%; Fig. 4c). Tumor cells were embedded in a fibrillary background matrix. Scattered, possibly entrapped ganglion cells were found in a subset of cases (56%). Anaplastic features like microvascular proliferation (95%), a high mitotic activity with an average mitotic count of up to 3.9 mitoses/mm^2^ (median 4 mitoses/mm^2^, range 0.4–6.7 mitoses/mm^2^, *n* = 12) and necrosis (32%) were common (Fig. 4b, d, e and Supplementary Table 2, online resource). Tumors were highly vascularized (Fig. 4f). Angiocentric arrangement mimicking ependymoma was seen in single cases (Fig. 4g). Tumor cell density was variable, in some cases showing a biphasic pattern with low-grade areas of moderate cell density (Fig. 4h), low mitotic activity and hyalinized vessels as well as areas of high cell density with numerous mitoses, microvascular proliferation and necrosis. Collectively, cell dense anaplastic areas were more frequently observed. Reticulin staining did not show an appreciable network in the tumor tissue apart from the vessels (Fig. 4i). Eosinophilic granular bodies and/or Rosenthal fibers were absent. Microcalcifications were seen in only one of the tumors. Immunohistochemistry revealed a diffuse positivity for synaptophysin with a weak or moderate cytoplasmic reactivity, while the tumor cell matrix showed a consistently strong staining (Fig. 5a). NeuN staining revealed scattered ganglion cells and only weak or negative staining of most tumor cells (Fig. 5b). Class III ß-tubulin and NSE were consistently positive in the tumors, although sometimes restricted to only a proportion of neoplastic cells (Fig. 5c and d). GFAP staining showed strong positivity of the tumor cell matrix with variable cytoplasmic reactivity between the cases and/or different areas within the same tumor (Fig. 5e). Olig2 was usually strongly expressed (Fig. 5f). MAP2 was positive (Fig. 5g), except in areas suggestive of hypoxia. CD34 expression was restricted to the vessels (Fig. 5h). Ki-67 labeling index was variable between different cases as well as within different areas of the same tumor. Lower grade areas showed only few positive nuclei while anaplastic areas showed a Ki-67 of up to 26% on average (median up to 20%, range 5–65%; Fig. 5i and Supplementary Table 2, online resource). Immunohistochemical ATRX expression was lost in 11 of 13 cases (85%) as described above. Detailed description of histological and immunohistochemical findings can be viewed in the supplement (Supplementary Table 2 and Supplementary Fig. 2, 3, 4, 5, 6, 7, 8, 9, 10, 11, 12, 13, 14, 15, 16, 17, 18, online resource).

### Clinical data indicate aggressive tumors that typically occur in children or young adults

**Fig. 6:**
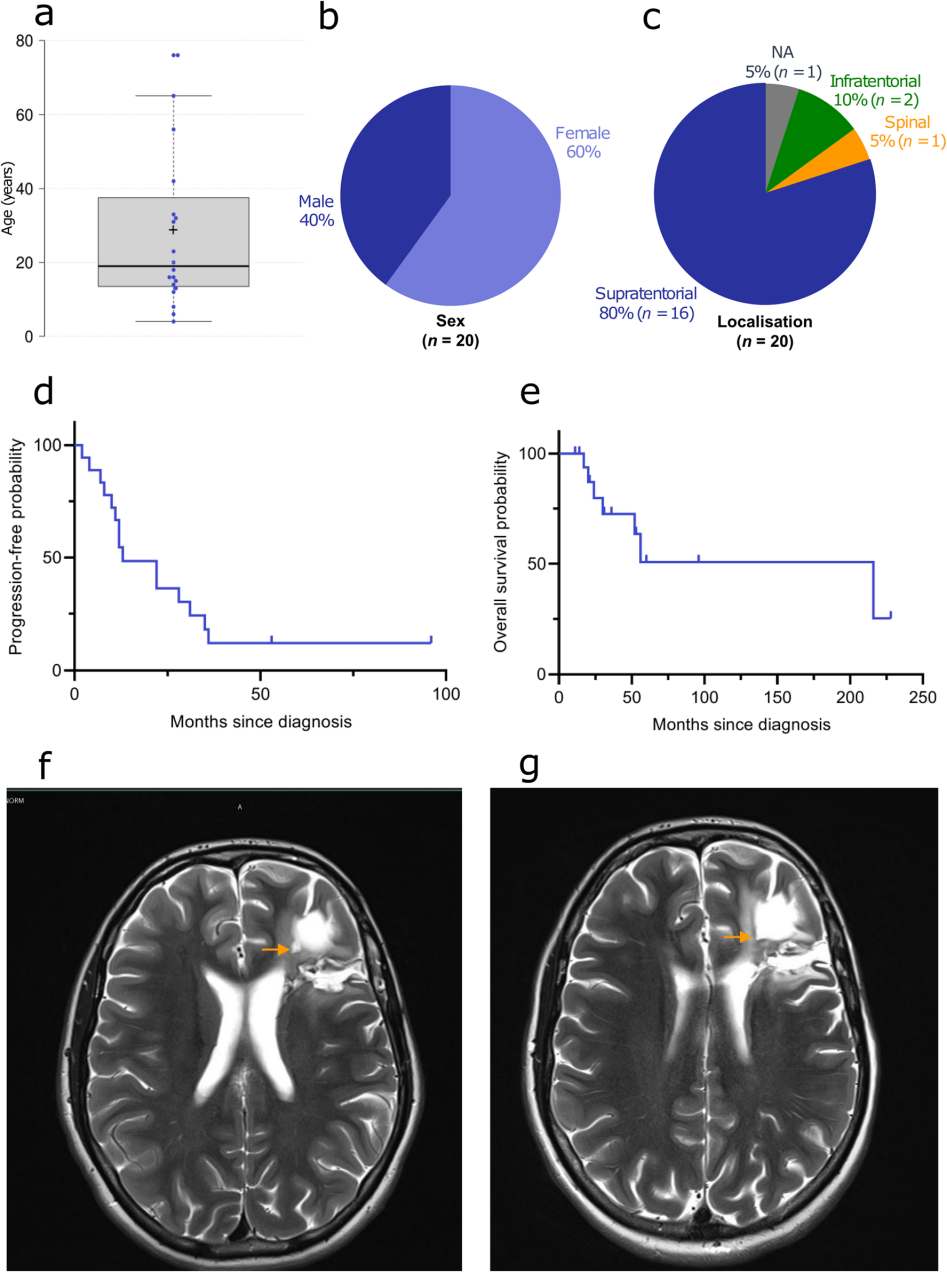
Clinical features of the investigated cohort. Age at diagnosis of the patients with a median age of 19 years (**a**), patient sex distribution (**b**) and distribution of tumor location (**c**). Progression-free (**d**) and overall survival (**e**) of the 18 patients from the investigated cohort for whom follow-up data were available. MRI (axial, T2) performed 12 months after surgery showing a small enhancing nodule along the cavity of resection (**f**). No sign of progression after 37 months of Larotrectinib (axial, T2; g)

Analysis of available clinical information revealed a mean age of the patients at the time of diagnosis of 28.8 years (median age: 19 years, range: 4–76 years, *n* = 20). Notably, 75% of patients were younger than 34 years at the time of diagnosis (Fig. 6a). The cohort consisted of 12 female patients and 8 male patients, resulting in a sex ratio of 2:3 (male:female; Fig. 6b; not significant). Data on tumor location was available in 19 of 20 of the cases (Fig. 6c). While 18 of 19 tumors occurred in the brain (95%), one showed a spinal location. Most tumors (84%) were found in the supratentorial compartment with only two cases occurring infratentorially (11%). Initial diagnosis of the tumors was very variable with some cases having a descriptive diagnosis (Supplementary Table 1, online resource). Most frequent tumor category was high-grade glioma (*n* = 8) including glioblastoma (*n* = 4) and anaplastic oligodendroglioma (*n* = 2). A proportion of tumors were described as ependymoma or clear cell ependymoma (*n* = 4). Only a subset of cases was diagnosed as glioneuronal tumor (*n* = 3).

Survival data was available for 18 of 20 patients. 15 patients experienced tumor recurrence after an average time of 22.9 months (median: 12.5, range: 2–53). 11 of 18 patients (61%) were alive at last follow-up with a mean follow-up time of 57.4 months (median: 33, range: 11–228). Seven patients (39%) have deceased with a mean survival time of 59.3 months (median: 30.5, range: 17–228; Fig. 6d, e).

From the available clinical data only one patient is known who received targeted NTRK inhibition (larotrectinib) after first recurrence, with no sign of progression on MRI after 37 months of ongoing therapy (Fig. 6f, g). Clinical data are summarized in the supplement (Supplementary Table 1, online resource).

## Discussion

Here, we describe a novel rare type of tumor that is characterized by a distinct epigenetic pattern and recurrent gene fusions mainly involving the *NTRK* gene family. *ATRX* alterations as well as typical morphological characteristics of isomorphic tumor cells with condensed nuclei and anaplastic features are additional unifying pattern of tumors within this group.

A first key finding of this study relates to the high frequency of oncogenic fusions detected in this series. While all tumors analyzed harbored a rearrangement involving different receptor tyrosine-kinases, most cases were found to show structural variants targeting *NTRK1-3*. In particular *NTRK2* was found with numerous different fusion partners. Contrary to previous reports [29], rearrangements involving *NTRK2* were not seen predominantly in the pediatric setting. In line with previous findings, all NTRK gene family fusions involved the 5′ end of the fusion partner and the 3′ end of NTRK preserving the tyrosine kinase domain resulting in constitutive activation of TRK signaling which ultimately leads to tumor proliferation and resistance to apoptosis [7]. In addition to *NTRK1-3*, all remaining cases also showed potentially targetable gene fusions mainly affecting the MAPK pathway. This offers therapeutic opportunities for targeted inhibition. In particular NTRK inhibition has proven to be mostly well tolerated and effective, although data on primary CNS tumors is still limited in comparison to other tumor types [2, 13]. Based on these findings, larotrectinib and entrectinib have recently received entity-agnostic FDA approval for NTRK fusion-positive tumors. In our series only one patient received targeted NTRK inhibition (larotrectinib) at first recurrence, with no signs of progression by MRI after 37 months of ongoing therapy.

Another important finding of this study was the high number of *ATRX* alterations detected in this series, which not only expands the spectrum of tumor types showing a recurrent loss of *ATRX* expression but also highlights a valuable diagnostic marker in particular in comparison to glioneuronal tumors such as extraventricular neurocytoma, diffuse leptomeningeal glioneuronal tumor or the recently described group of glioneuronal tumors driven by different kinase-fusions [25, 28]. *ATRX* is recurrently mutated in IDH-mutant astrocytoma [14], H3 K27-altered diffuse midline glioma [22], H3 G34-mutant diffuse hemispheric glioma [12] and high-grade astrocytoma with piloid features (HGAP) [20]. In particular HGAP would be one of the most probable differential diagnoses of this new group of tumors in terms of histopathology and molecular profile. These tumors, in addition to MAPK alterations, are usually characterized by anaplastic features and frequently harbor homozygous deletions of *CDKN2A/B* as well as loss of ATRX expression [20]. However, piloid features such as eosinophilic granular bodies and/or Rosenthal fibers typically found in HGAP were absent in the present series. In addition, the high frequency of gene fusions in particular rearrangements involving the *NTRK* gene family as observed in this study is not very typical for HGAPs. Further differences apply to the tumor location. While HGAP is mainly located infratentorially (74%), tumors within our cohort were found most frequently in the supratentorial compartment [20]. Further differential diagnoses include infant-type hemispheric glioma and glioneuronal tumors driven by different kinase-fusions that are characterized by the presence of RTK fusions as well including the *NTRK* family gene [6, 10, 25]. However, these tumors typically arise in early childhood / in a pediatric setting and do not show *ATRX* alterations or homozygous deletions of *CDKN2A/B* [6, 10, 25, 28].

An important issue raised in the context of the histopathological and molecular data generated here is where these tumors fit best into the current WHO CNS tumor taxonomy. Given the heterogeneous morphology of the tumors with a wide range of original diagnoses it seems difficult to classify them at first sight. However, since co-expression of different glial and neuronal markers have been characteristic for tumors within this series, it seems reasonable to classify them as ‘glioneuronal’, which also fits to the morphological appearance of the tumors.

While most glioneuronal tumors show a relatively benign clinical behavior and can often be cured by surgery when amenable to complete resection [4, 15], tumors within this novel group are characterized by a more aggressive biology. This is underlined by the clinical course (although follow-up data are limited), the anaplastic features as well as a high frequency of homozygous deletions of *CDKN2A/B* in these tumors. Future studies are needed to give more reliable prognostic information.

In summary, the identification of this novel type of glioneuronal tumor emphasizes once again the great benefit of molecular characterization in CNS tumor diagnostics and treatment. The high frequency of targetable fusion events found in theses tumors provides further significant opportunities for patient care. Given their molecular characteristics in addition to anaplastic features, we suggest the term glioneuronal tumor with *ATRX* alteration, kinase fusion and anaplastic features (GTAKA) to describe these tumors.

## Supplementary Information

Below is the link to the electronic supplementary material.

Supplementary file1 (PDF 8754 KB)

Supplementary file2 (XLSX 23 KB)

## Data Availability

The data that support the findings of this study are available from the corresponding author upon reasonable request.
