# Supplementary material for: Glioneuronal tumor with ATRX alteration, kinase fusion and anaplastic features (GTAKA): a molecularly distinct brain tumor type with recurrent NTRK gene fusions
Source: Acta Neuropathol. 2023 Mar 18;145(5):667–80. doi: 10.1007/s00401-023-02558-0 (PMC10119244; doi:10.1007/s00401-023-02558-0)
Supplement: Supplementary file 1 — Supplementary file1 (PDF 8754 KB) [file 401_2023_2558_MOESM1_ESM.pdf]

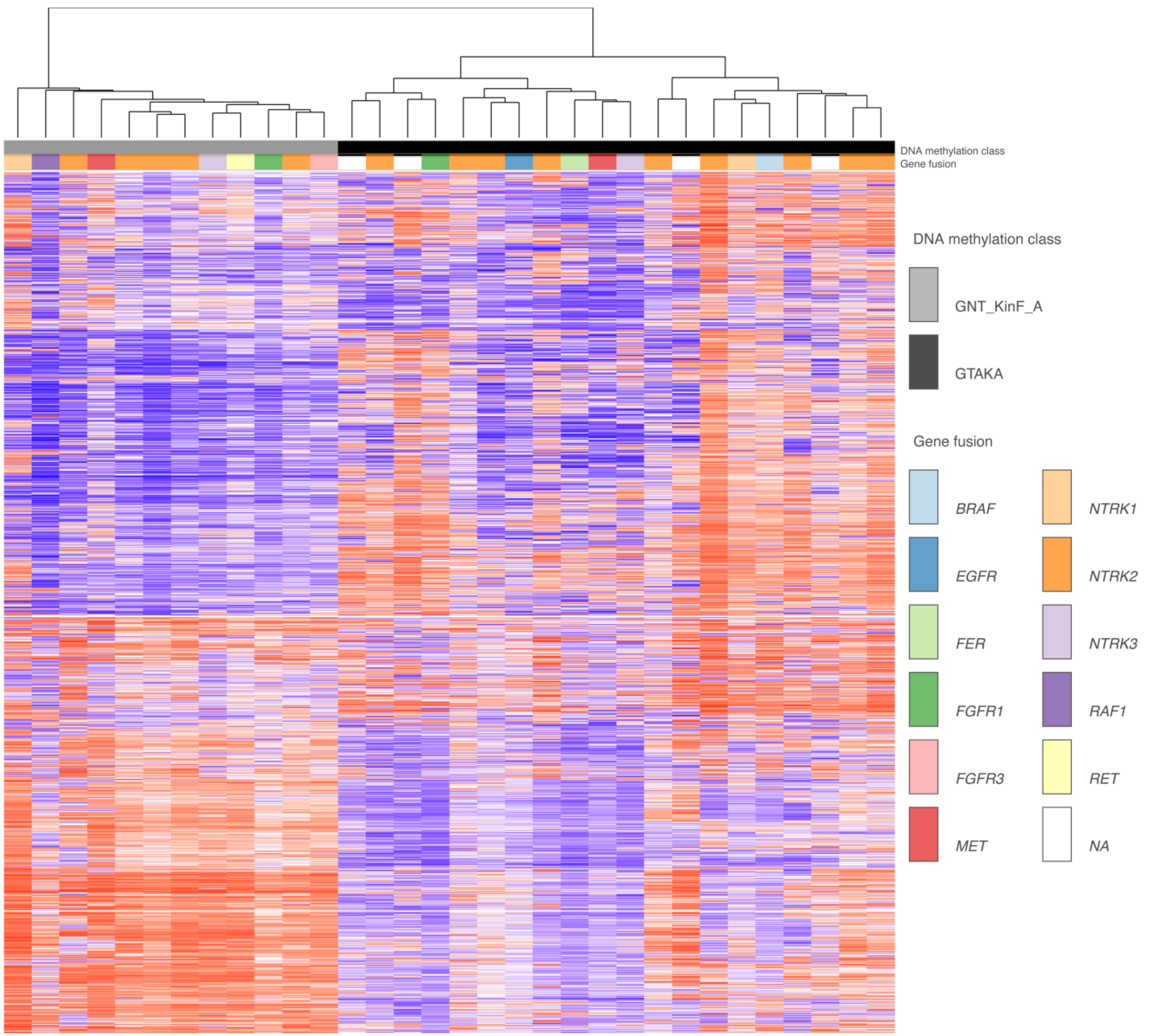

Suppl. Fig. 1: Unsupervised hierarchical clustering of DNA methylation profiles of GTAKA and GNT_KinF_A using the 10,000 most variably methylated probes.

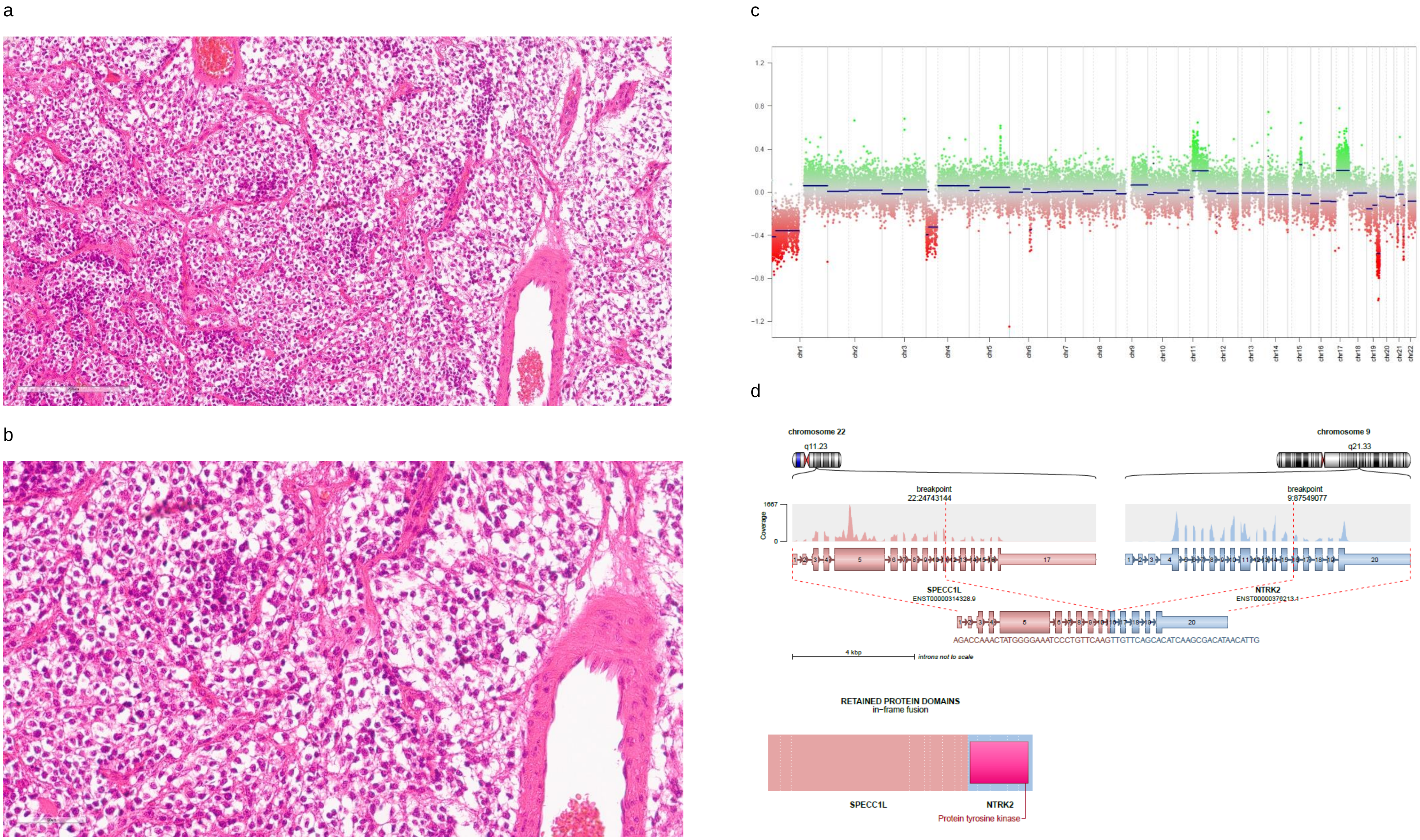

Suppl. Fig. 2: Representative H&E pictures (a, b), the copy-number profile derived from DNA methylation data (c) and a visualization of the gene fusion detected by RNA sequencing (d) of case #1 are presented.

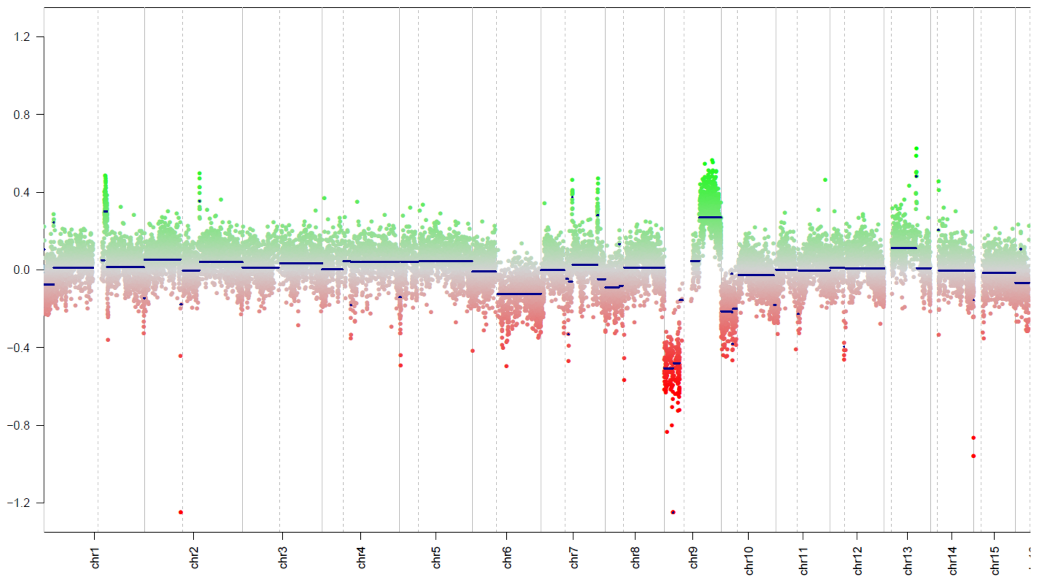

Suppl. Fig. 3: The copy-number profile derived from DNA methylation data of case #2 can be viewed. Histological workup and RNA-based fusion analysis was performed in Boston, MA, USA. Fusion analysis revealed a *SPECC1L::NTRK2* fusion (PMID: 32665022).

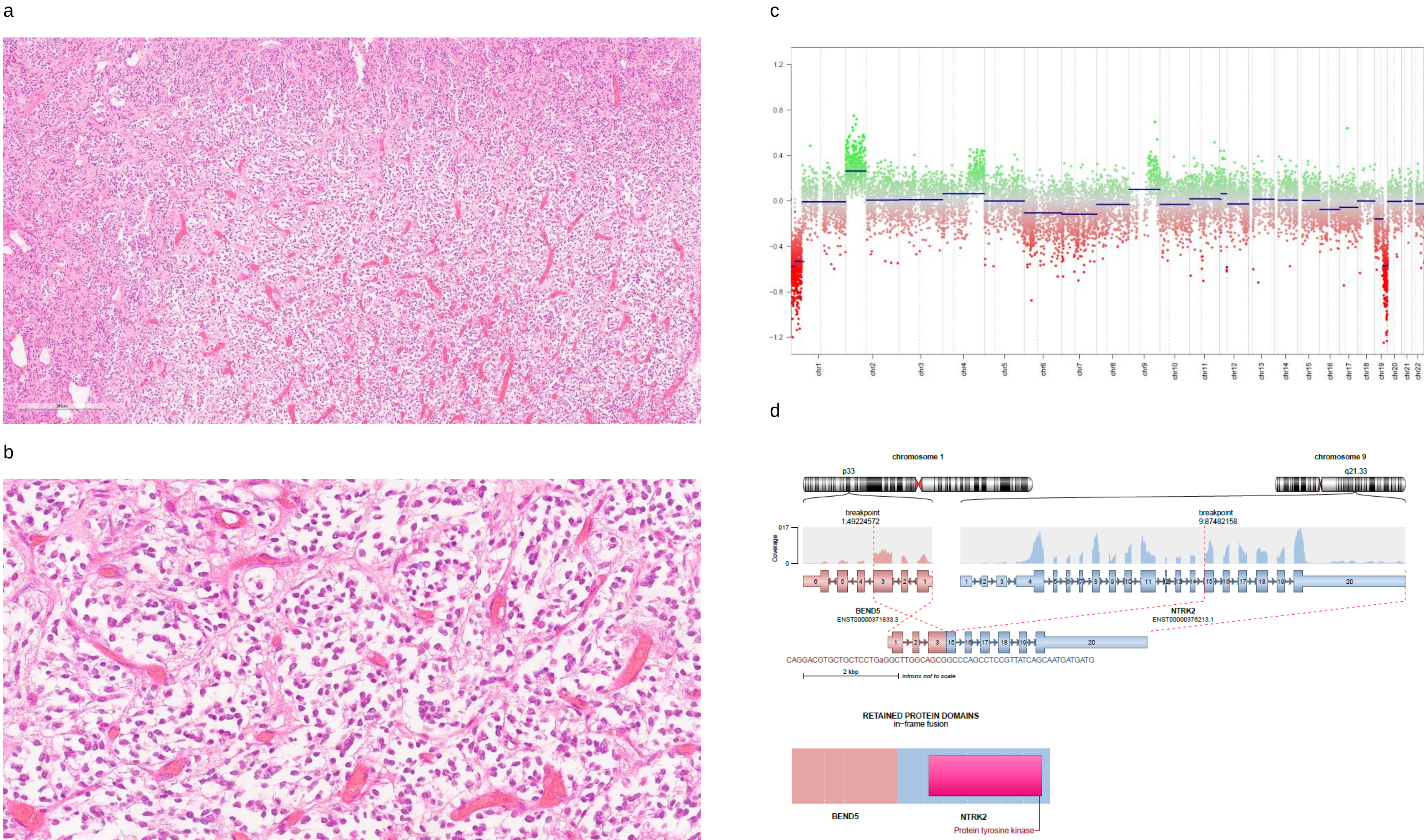

Suppl. Fig. 4: Representative H&E pictures (a, b), the copy-number profile derived from DNA methylation data (c) and a visualization of the gene fusion detected by RNA sequencing (d) of case #3 are presented.

a

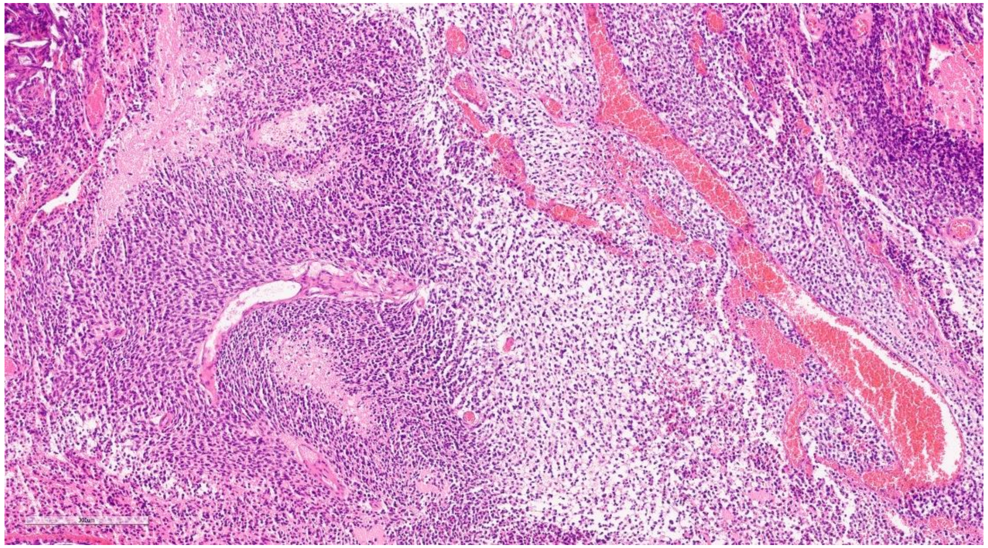

b

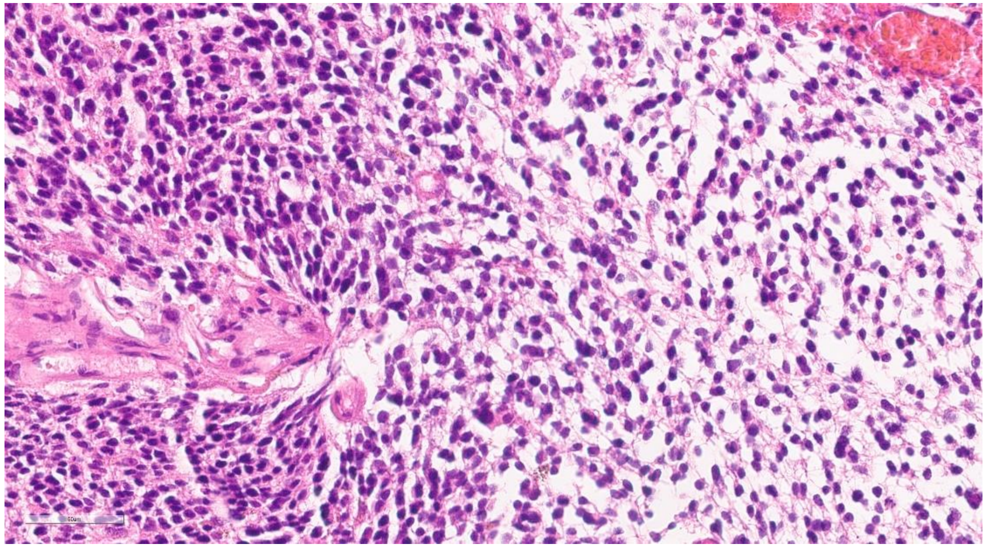

c

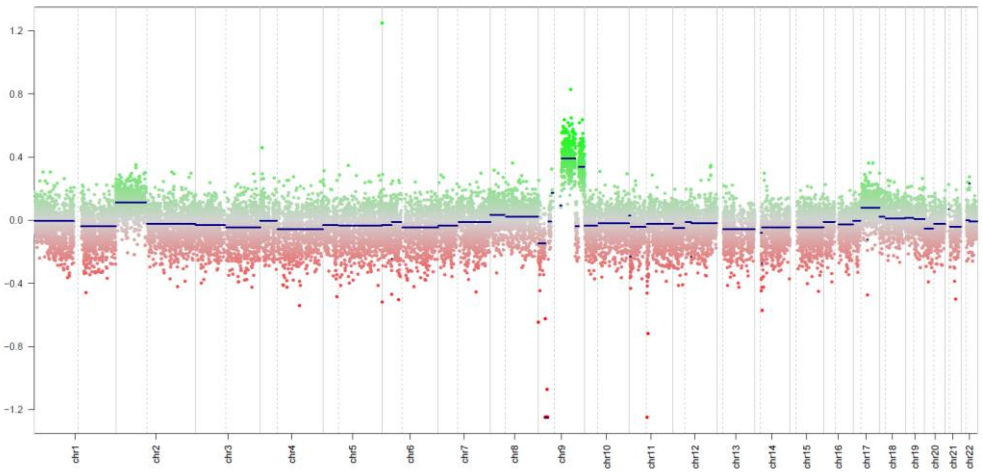

d

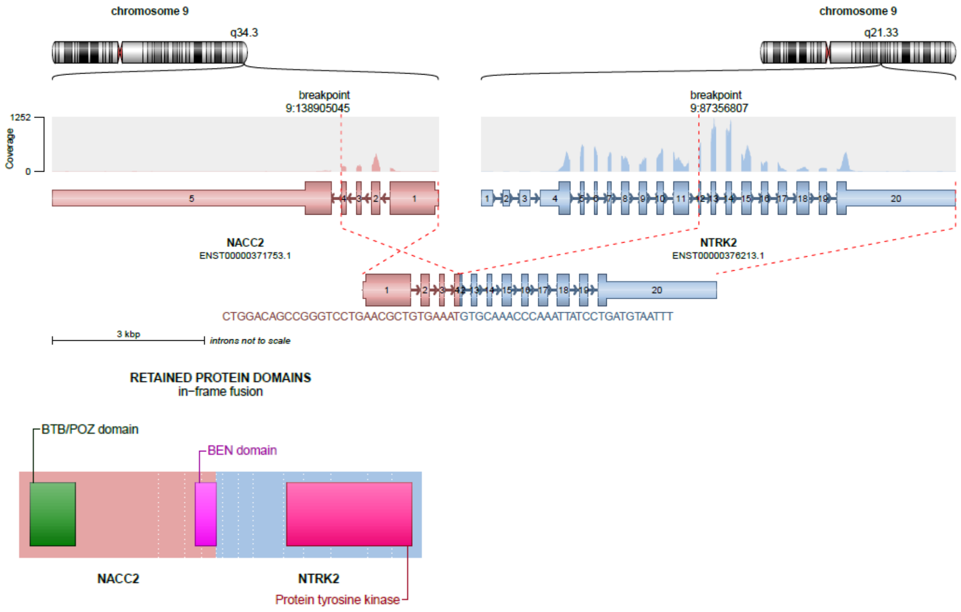

Suppl. Fig. 5: Representative H&E pictures (a, b), the copy-number profile derived from DNA methylation data (c) and a visualization of the gene fusion detected by RNA sequencing (d) of case #4 are presented.

a

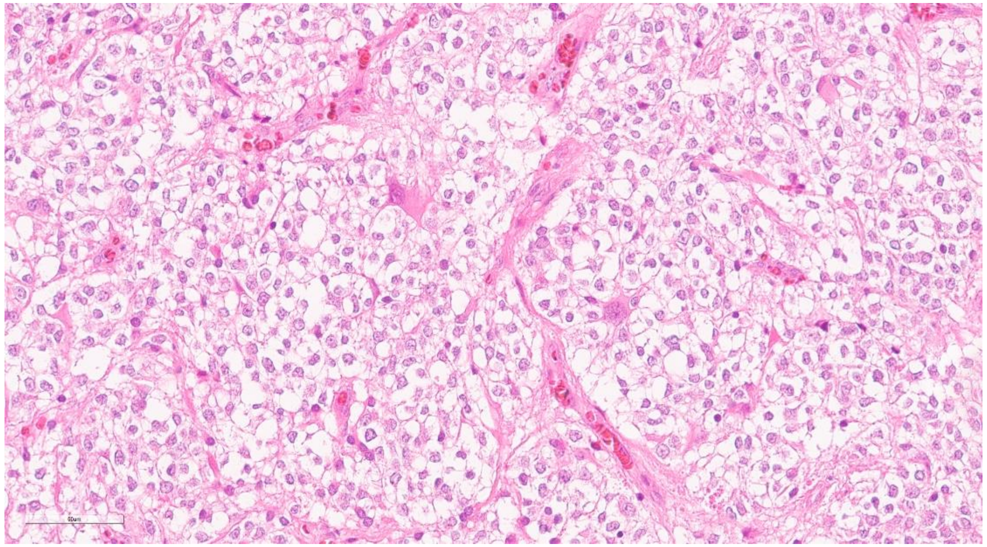

b

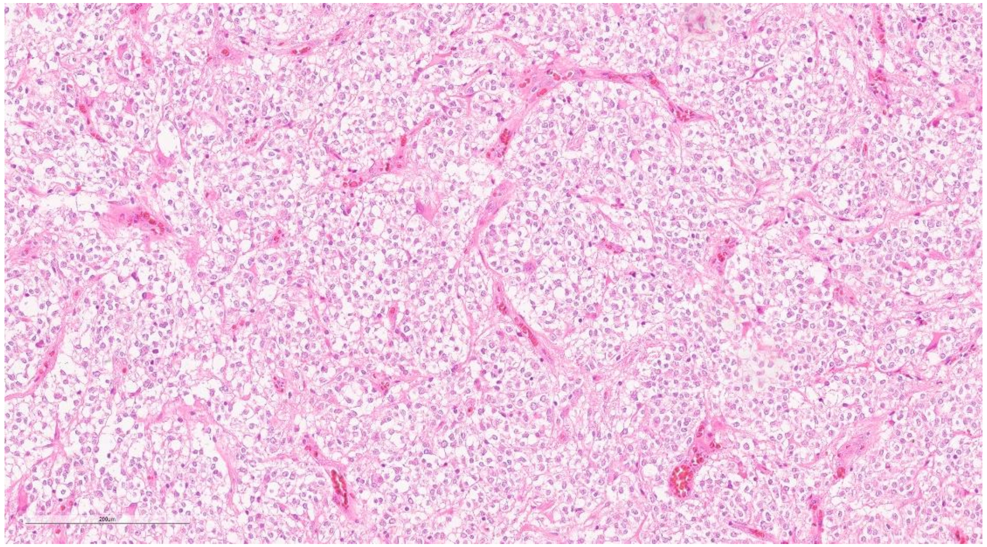

c

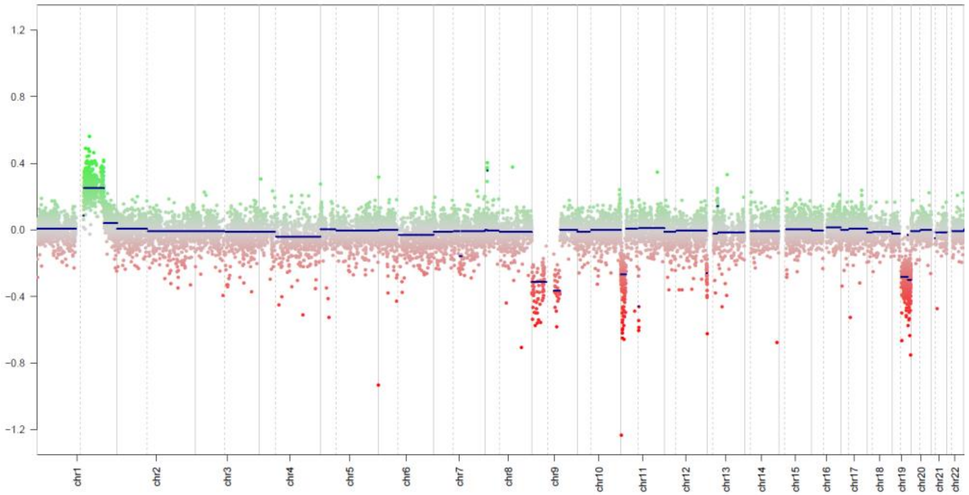

d

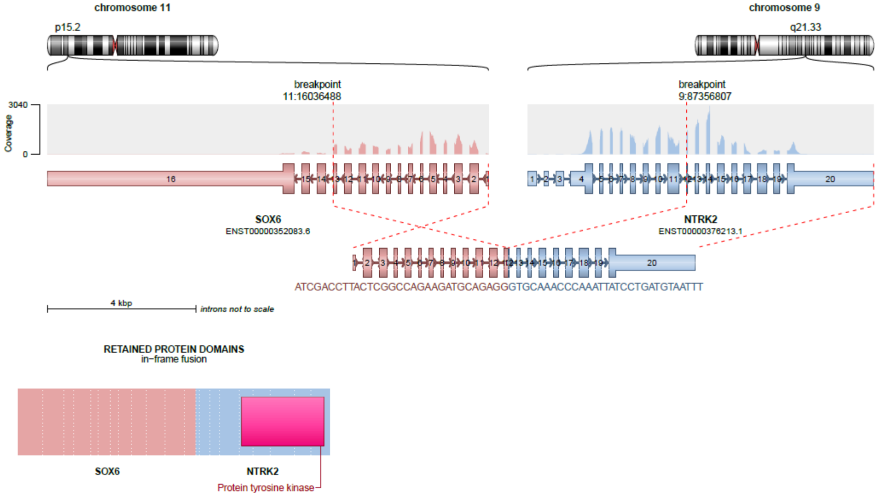

Suppl. Fig. 6: Representative H&E pictures (a, b), the copy-number profile derived from DNA methylation data (c) and a visualization of the gene fusion detected by RNA sequencing (d) of case #5 are presented.

a

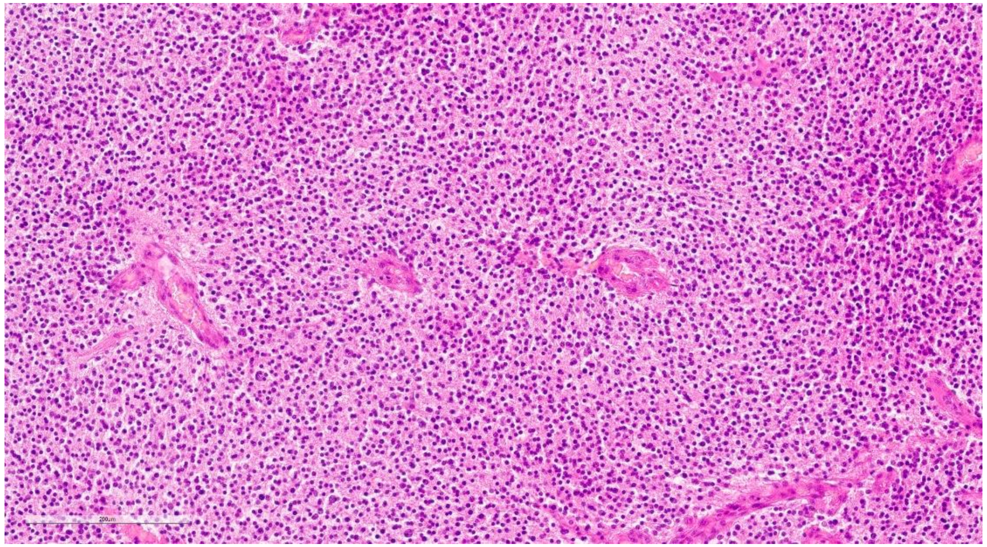

b

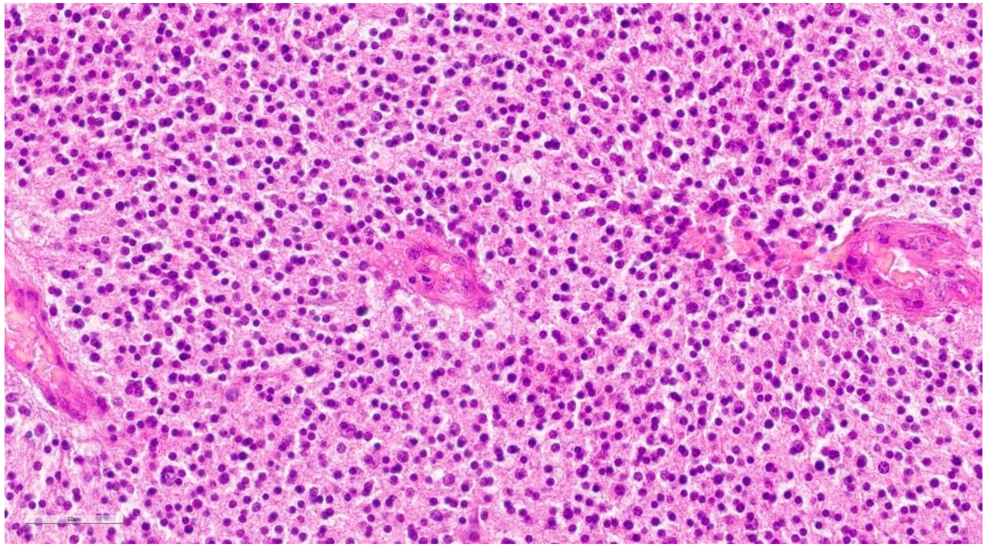

c

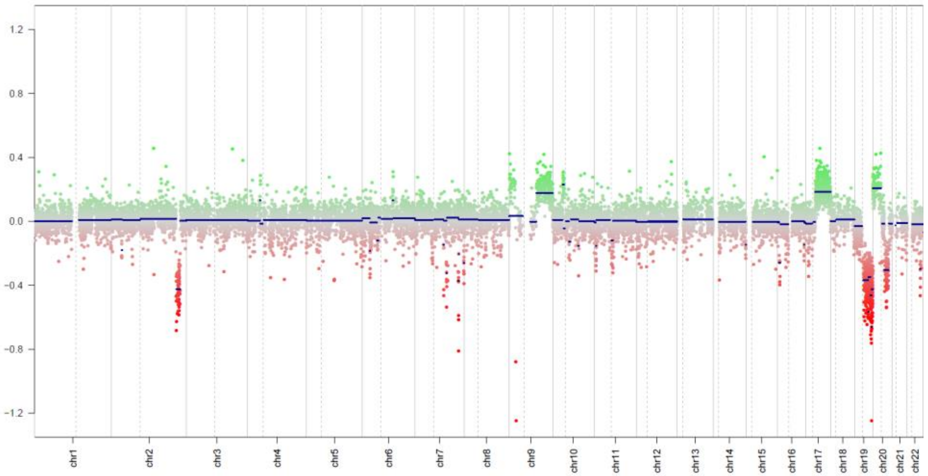

d

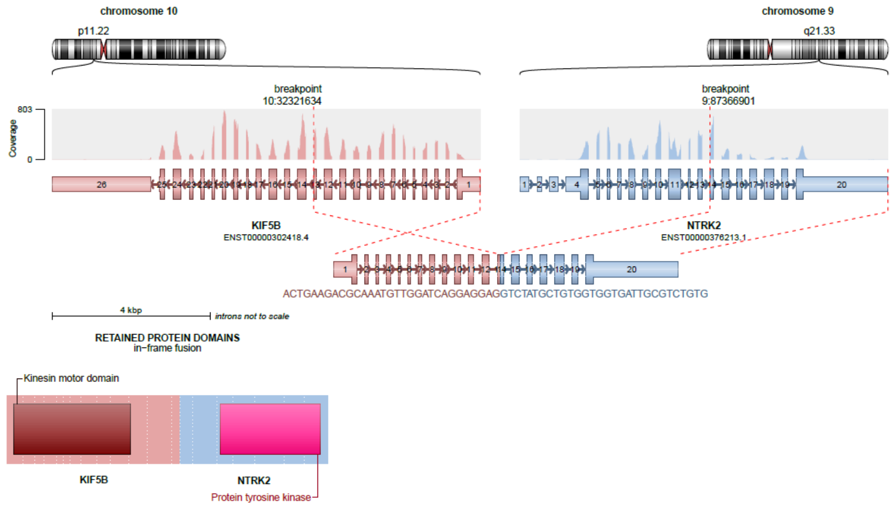

Suppl. Fig. 7: Representative H&E pictures (a, b), the copy-number profile derived from DNA methylation data (c) and a visualization of the gene fusion detected by RNA sequencing (d) of case #6 are presented.

a

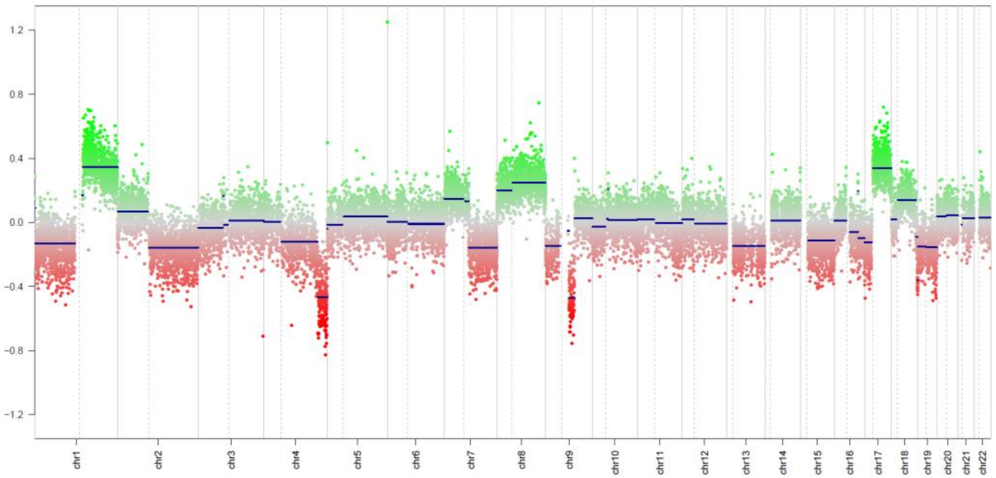

b

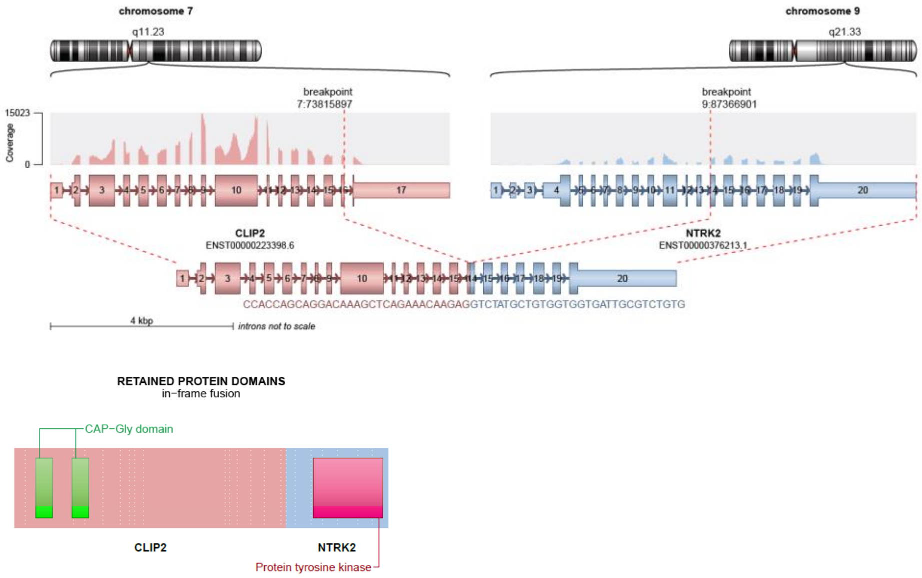

Suppl. Fig. 8: The copy-number profile derived from DNA methylation data (a) and a visualization of the gene fusion detected by RNA sequencing (b) of case #7 are presented. Histology was performed externally.

a

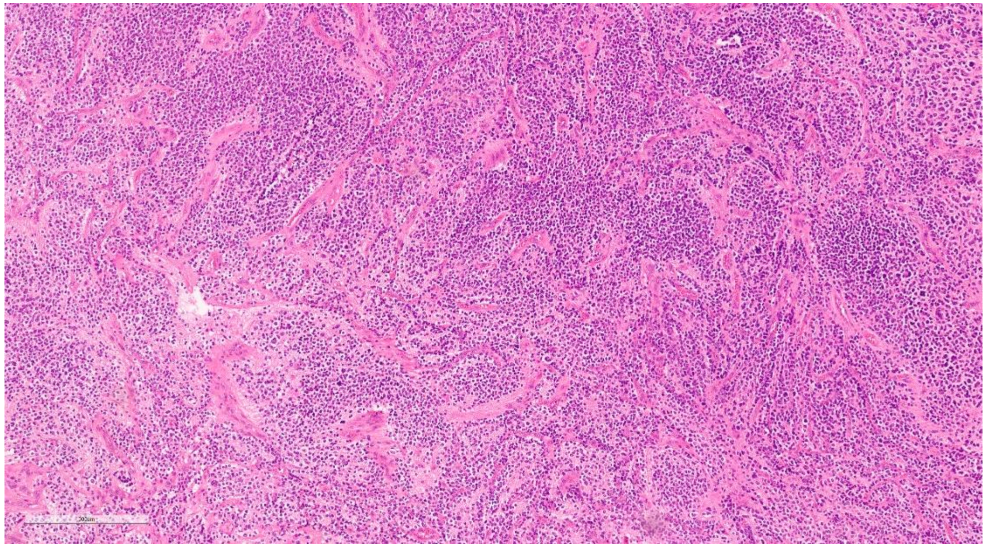

b

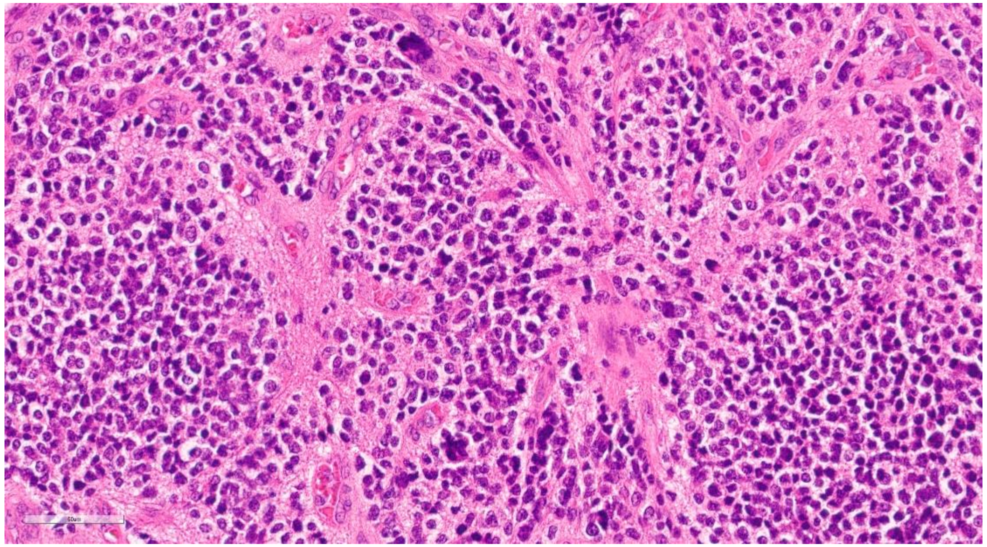

c

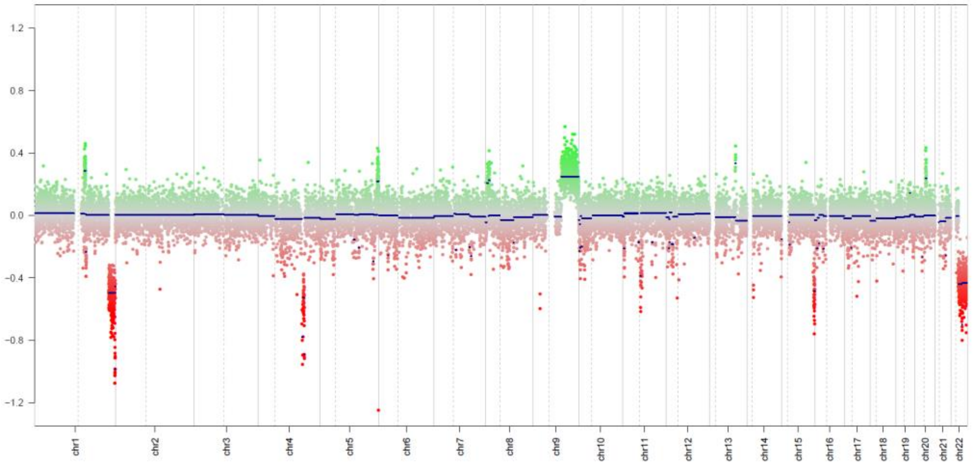

d

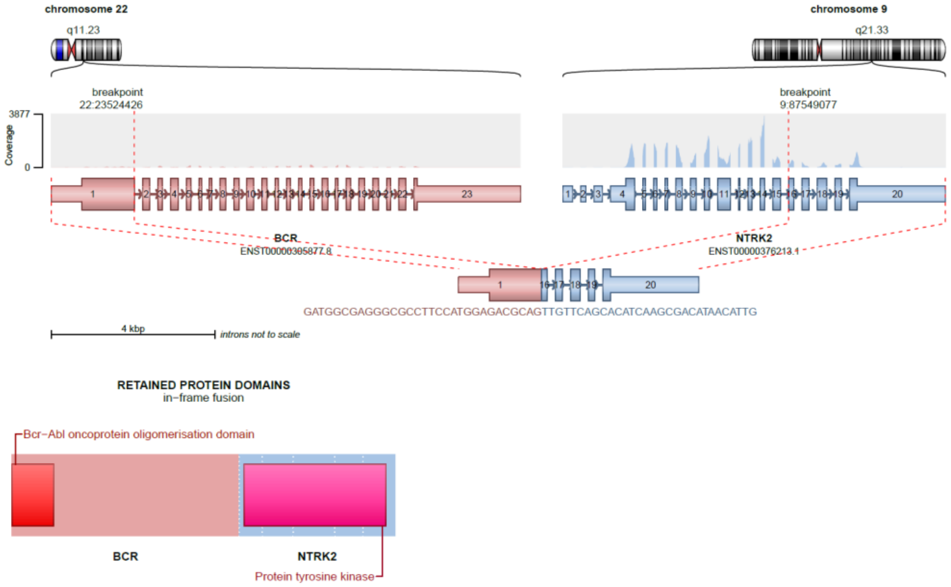

Suppl. Fig. 9: Representative H&E pictures (a, b), the copy-number profile derived from DNA methylation data (c) and a visualization of the gene fusion detected by RNA sequencing (d) of case #8 are presented.

a

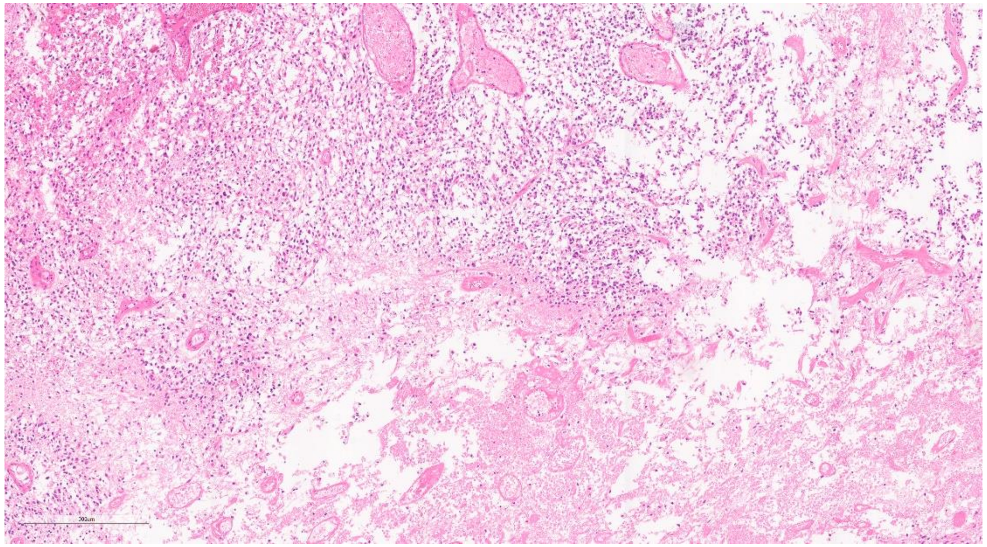

b

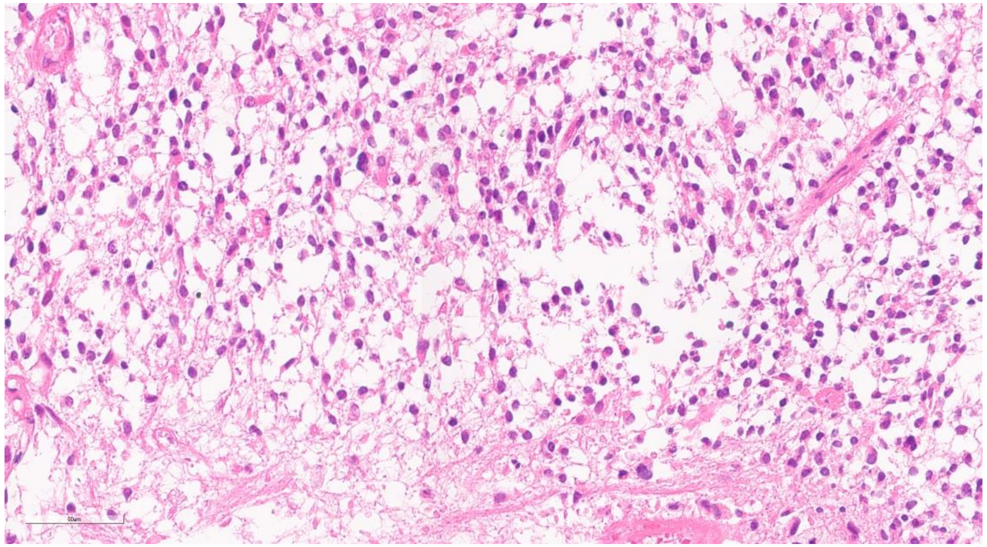

c

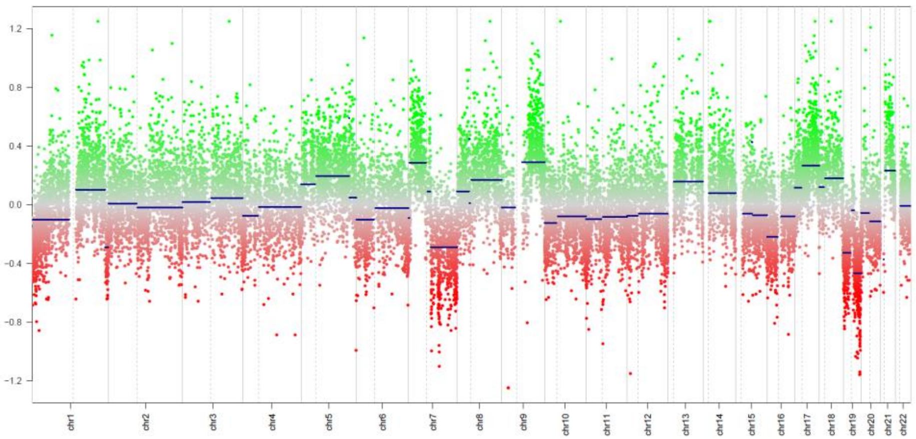

d

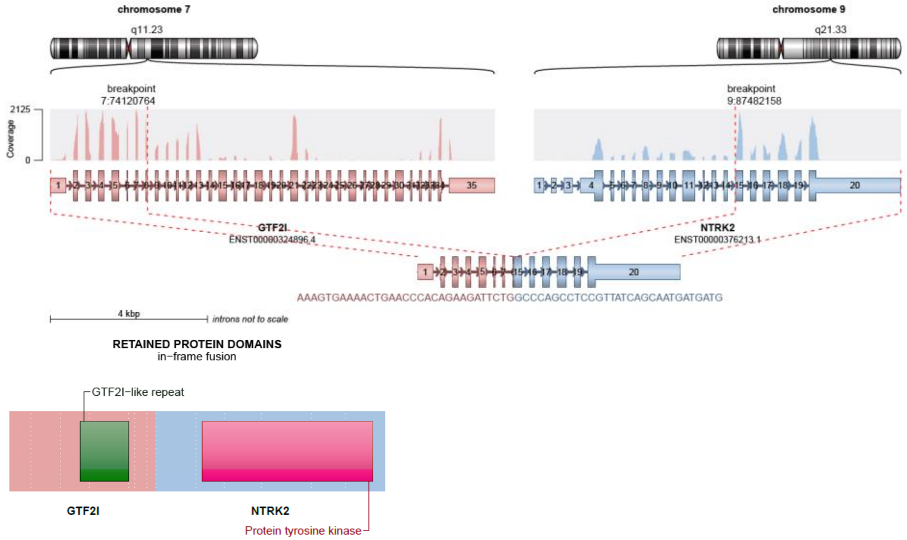

Suppl. Fig. 10: Representative H&E pictures (a, b), the copy-number profile derived from DNA methylation data (c) and a visualization of the gene fusion detected by RNA sequencing (d) of case #9 are presented.

a

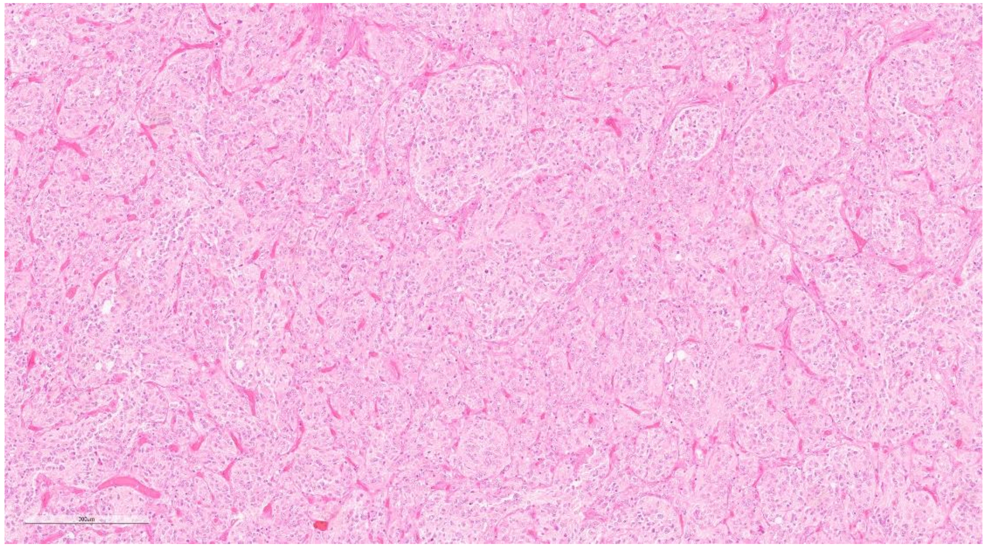

b

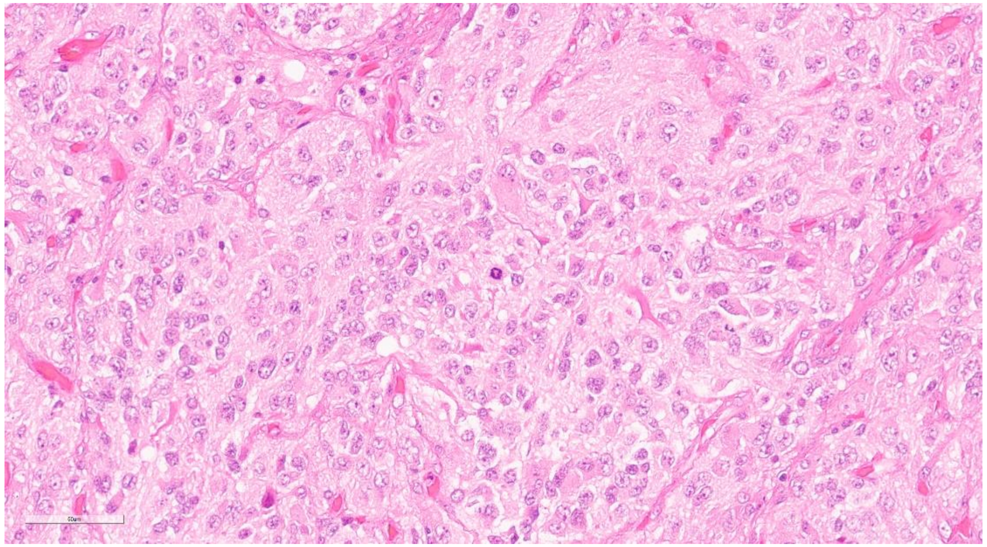

c

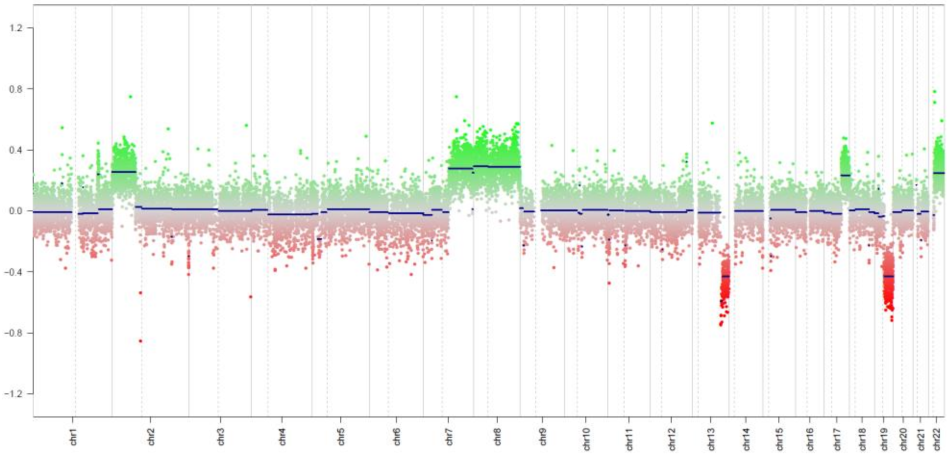

d

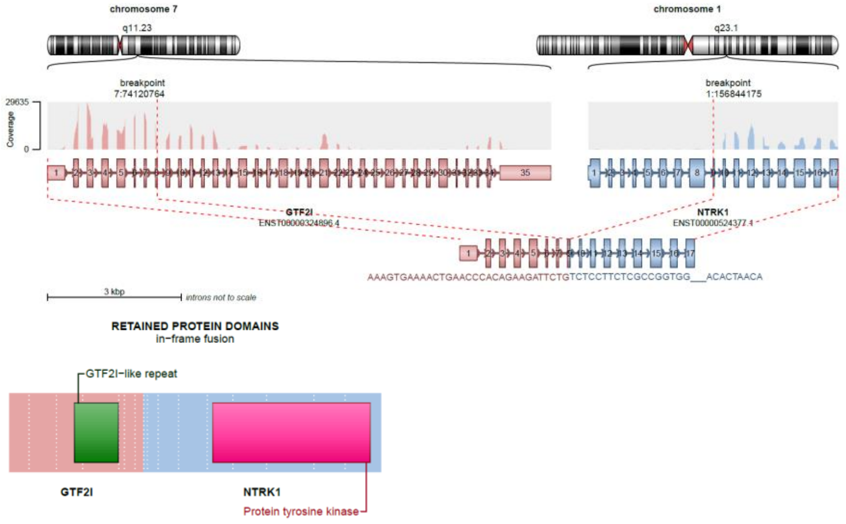

Suppl. Fig. 11: Representative H&E pictures (a, b), the copy-number profile derived from DNA methylation data (c) and a visualization of the gene fusion detected by RNA sequencing (d) of case #10 are presented.

a

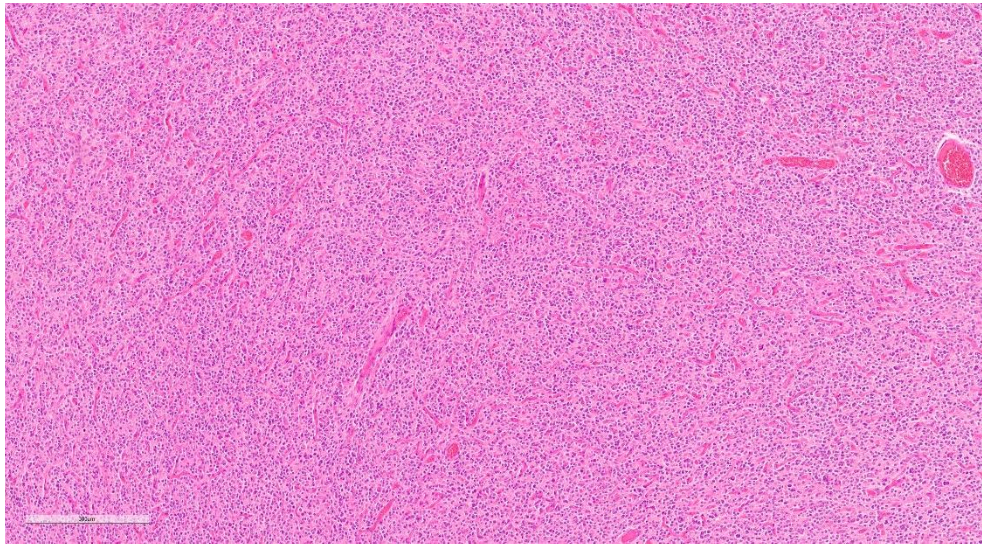

b

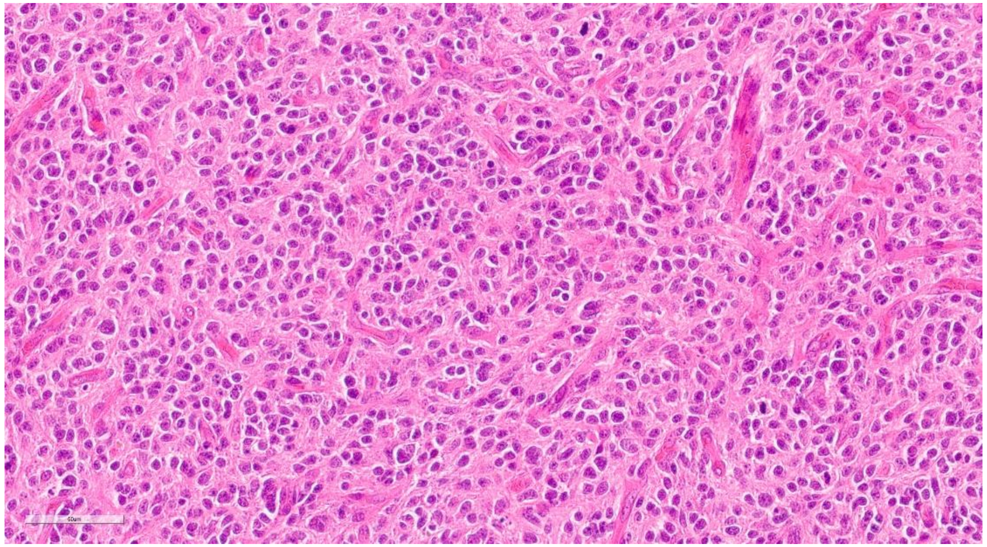

c

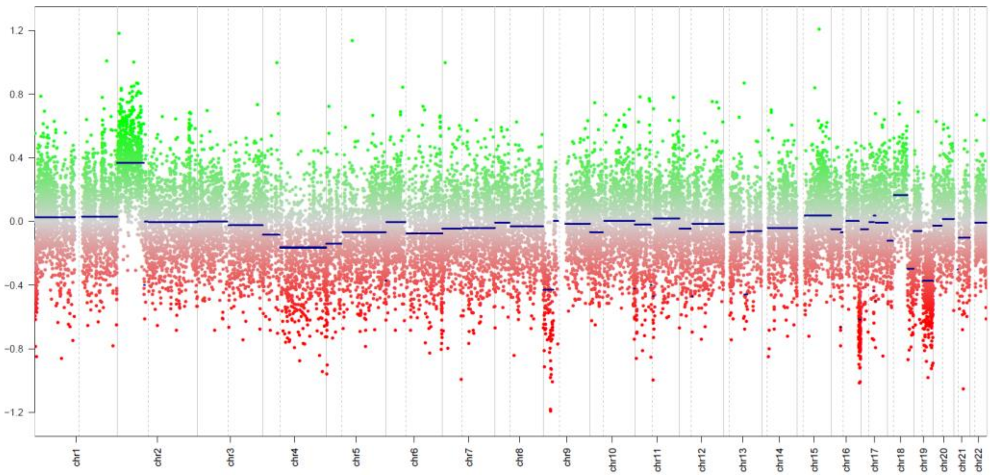

d

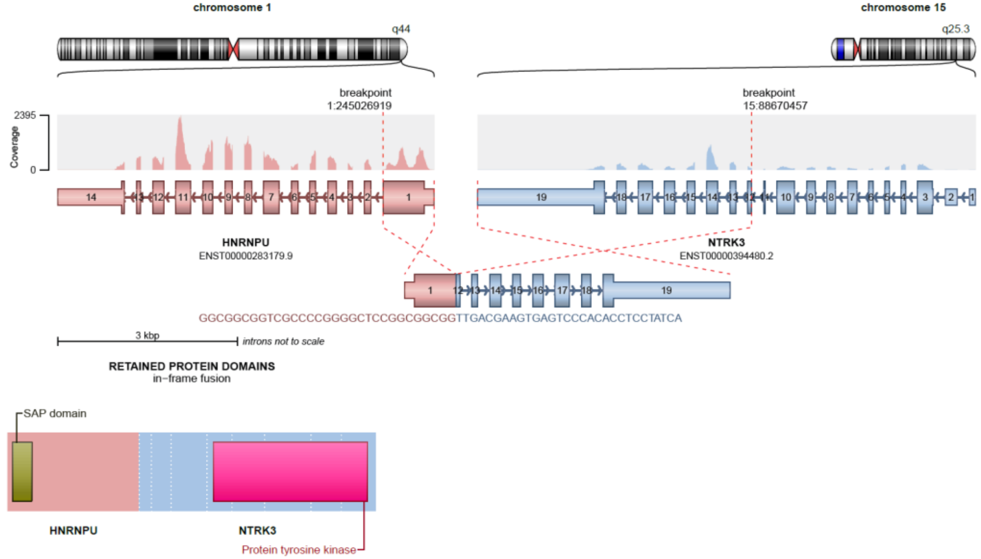

Suppl. Fig. 12: Representative H&E pictures (a, b), the copy-number profile derived from DNA methylation data (c) and a visualization of the gene fusion detected by RNA sequencing (d) of case #11 are presented.

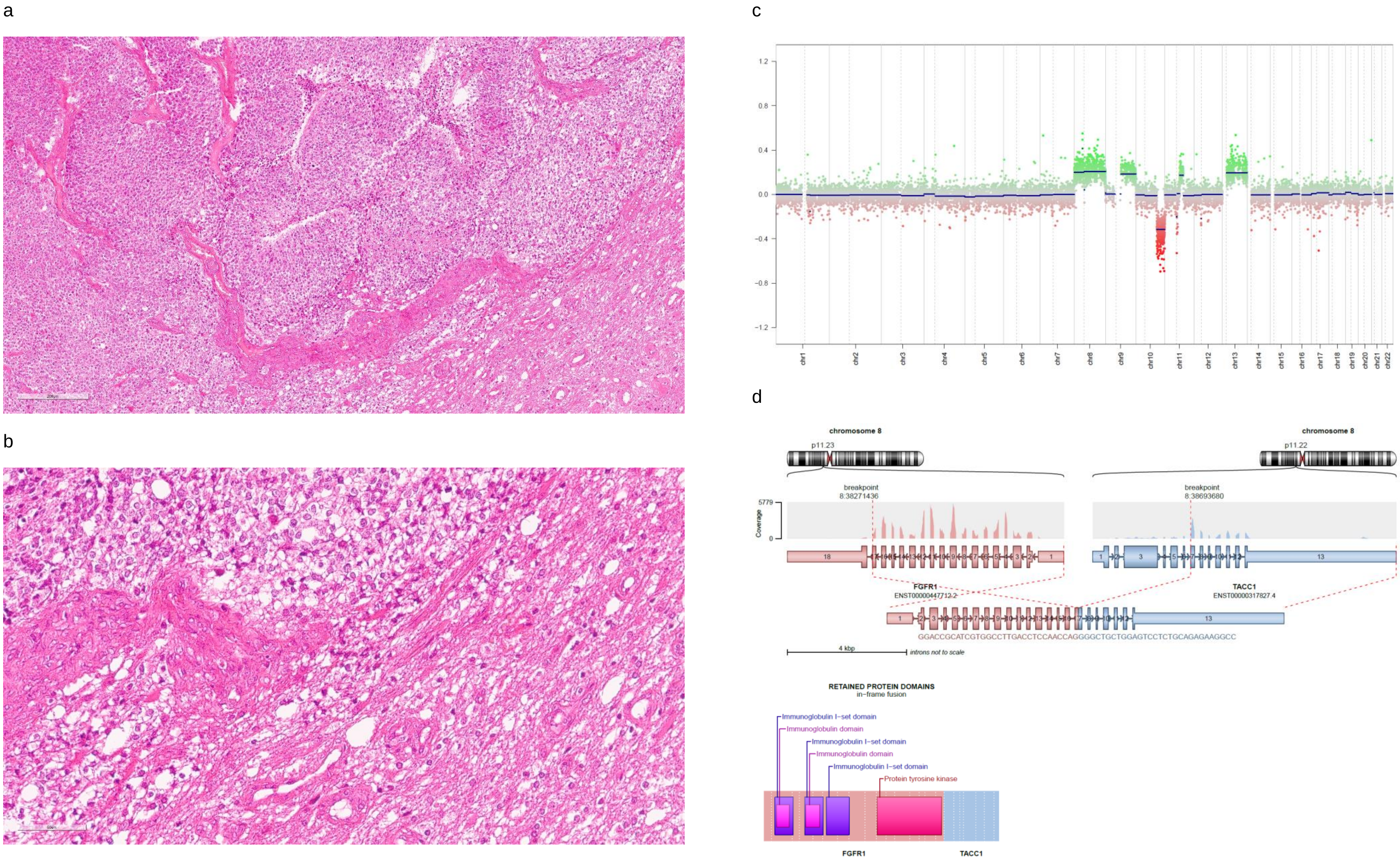

Suppl. Fig. 13: Representative H&E pictures (a, b), the copy-number profile derived from DNA methylation data (c) and a visualization of the gene fusion detected by RNA sequencing (d) of case #12 are presented.

a

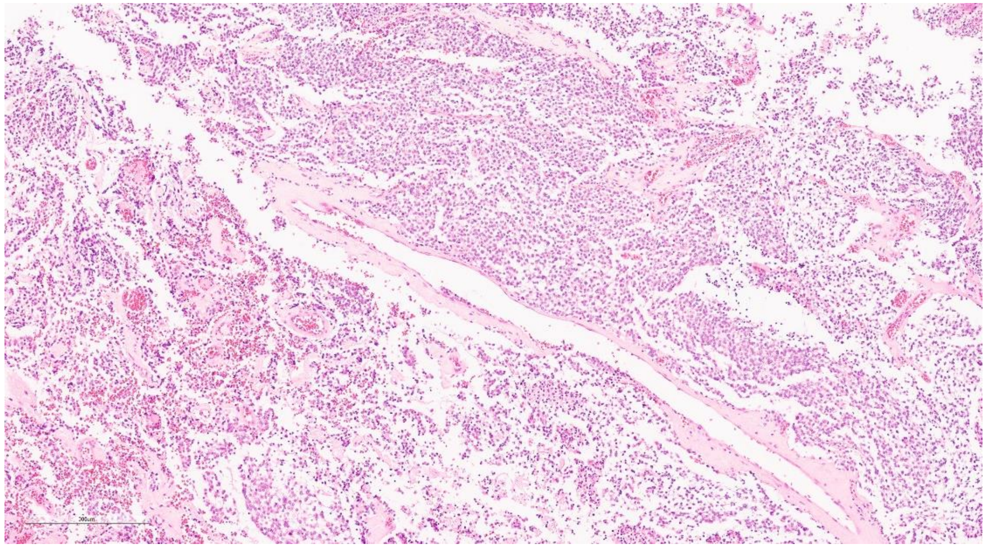

b

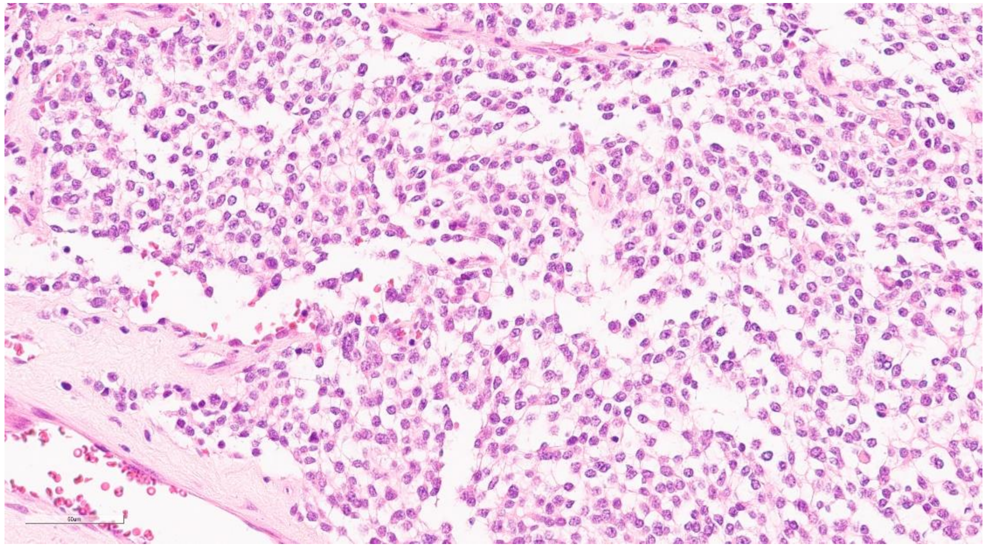

c

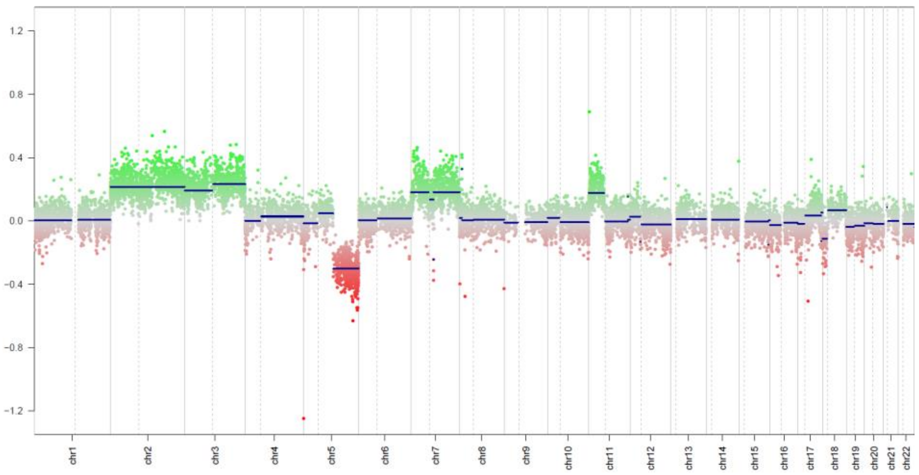

d

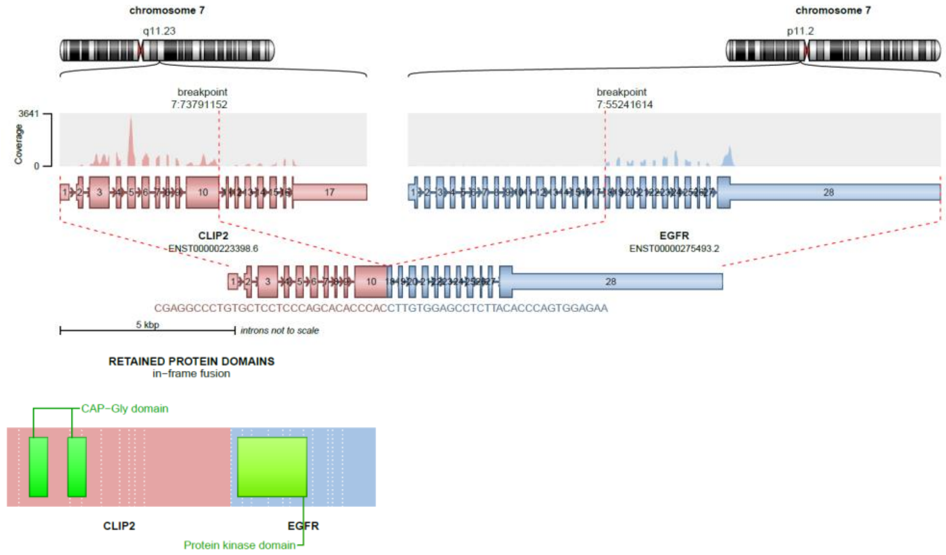

Suppl. Fig. 14: Representative H&E pictures (a, b), the copy-number profile derived from DNA methylation data (c) and a visualization of the gene fusion detected by RNA sequencing (d) of case #13 are presented.

a

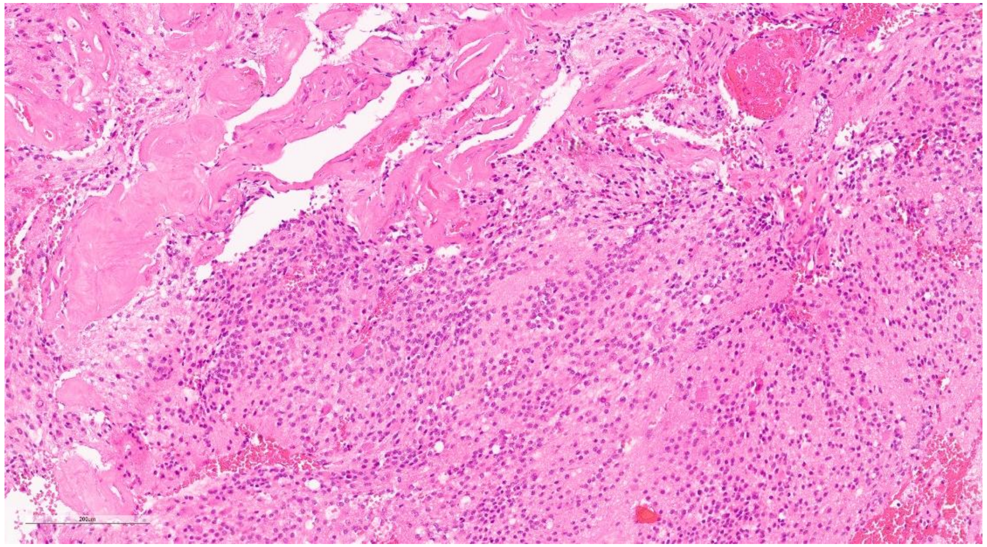

b

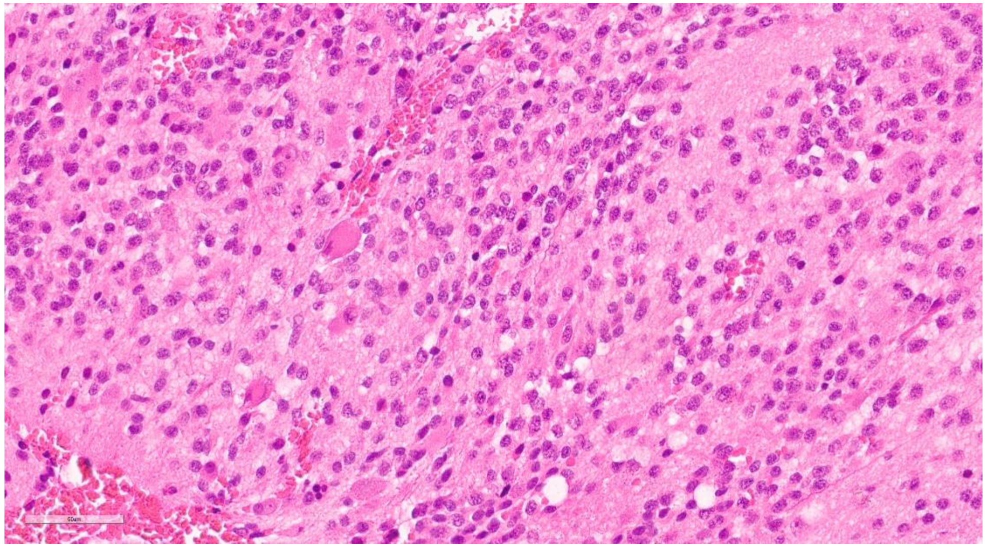

c

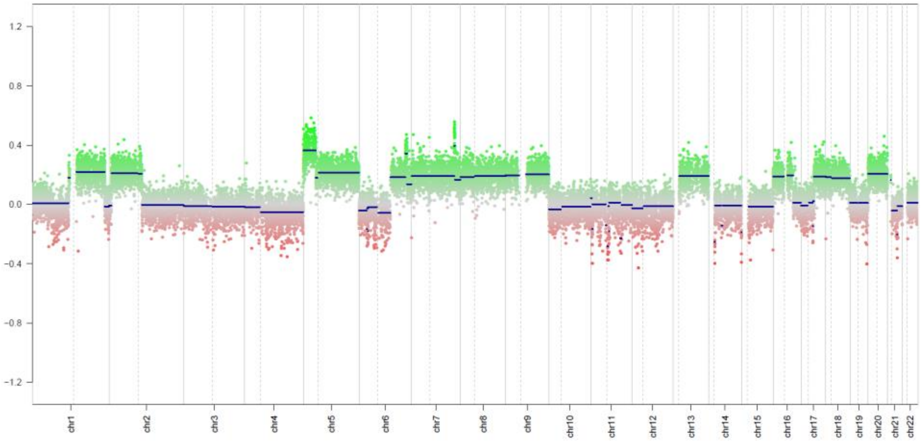

Suppl. Fig. 15: Representative H&E pictures (a, b) and the copy-number profile derived from DNA methylation data (c) of case #14 are presented. Fusion analysis by Archer Fusionplex Solid tumor panel (Copenhagen, Denmark). Detection of a *KIAA1549::BRAF* fusion.

a

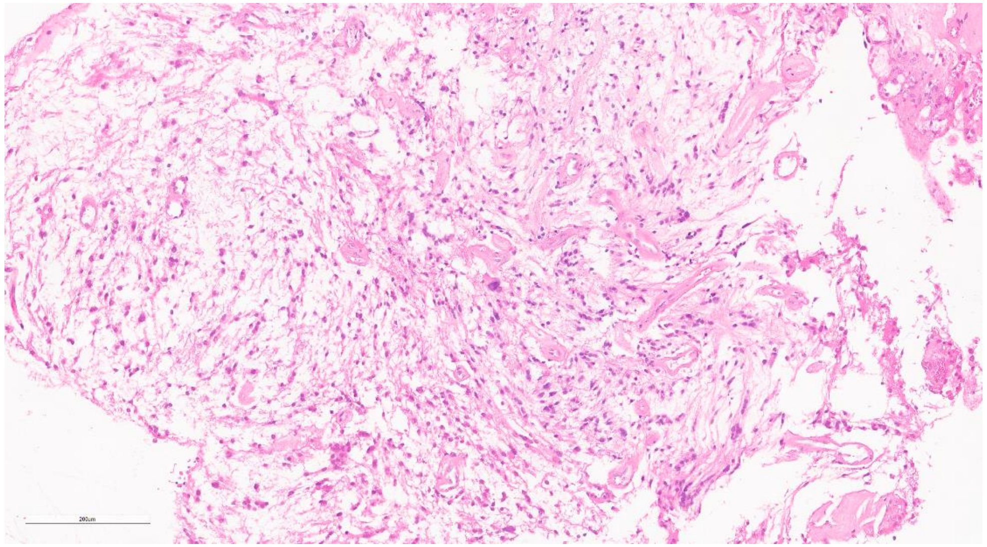

b

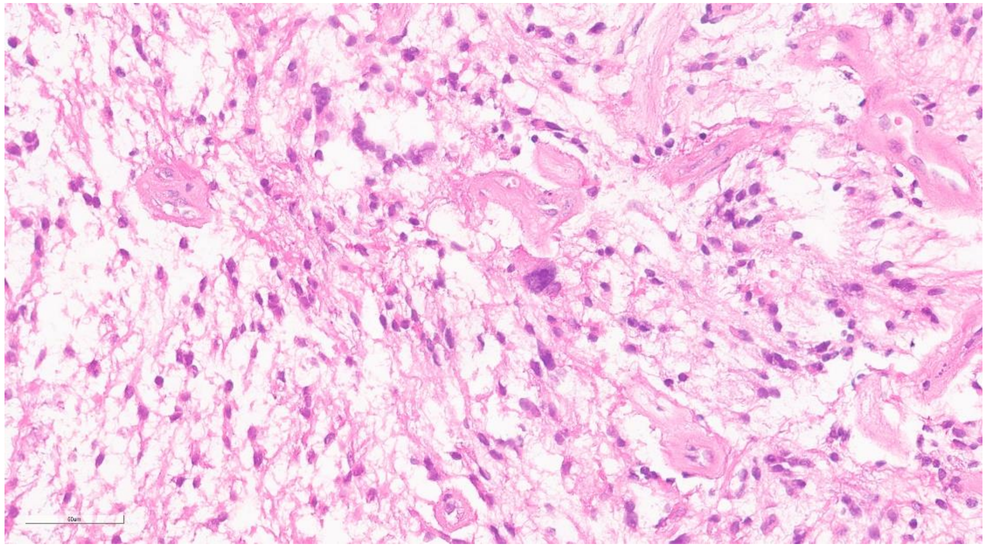

c

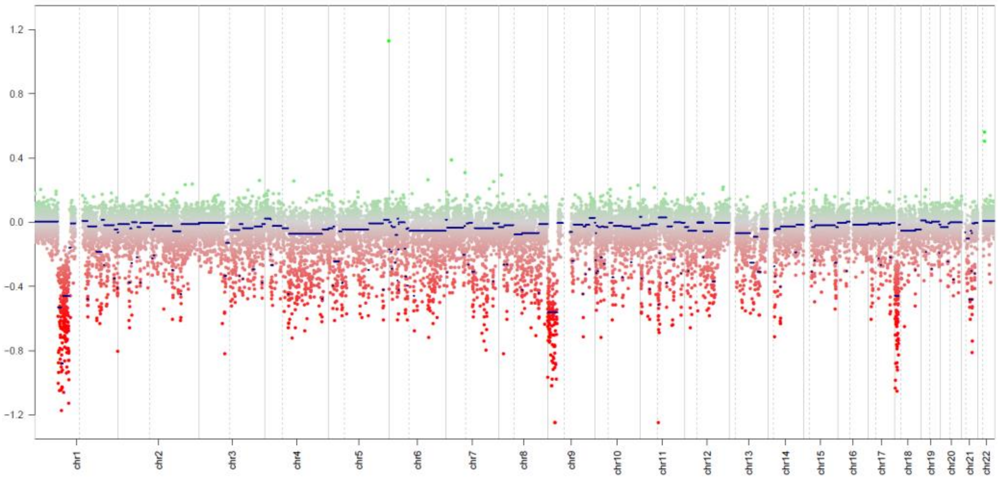

d

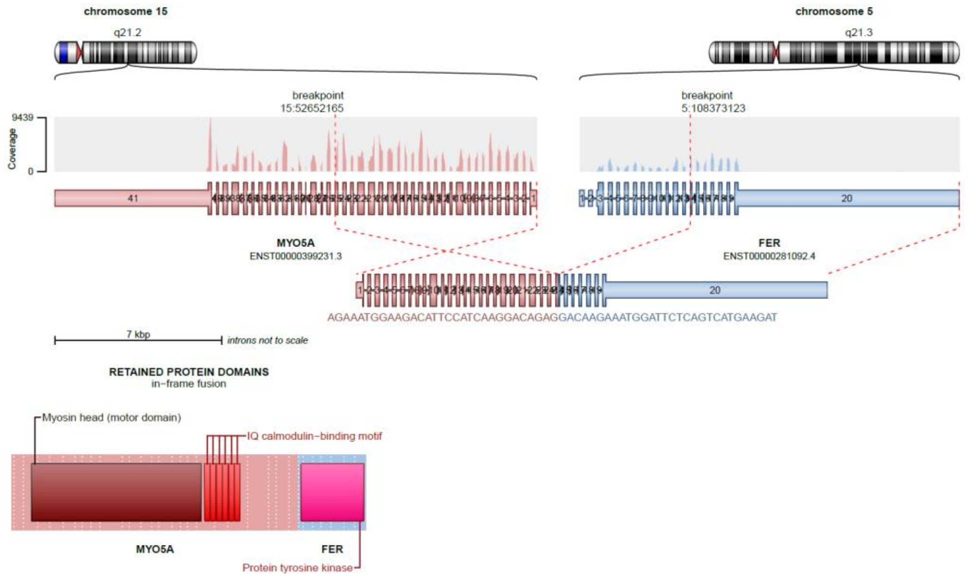

Suppl. Fig. 16: Representative H&E pictures (a, b), the copy-number profile derived from DNA methylation data (c) and a visualization of the gene fusion detected by RNA sequencing (d) of case #15 are presented.

a

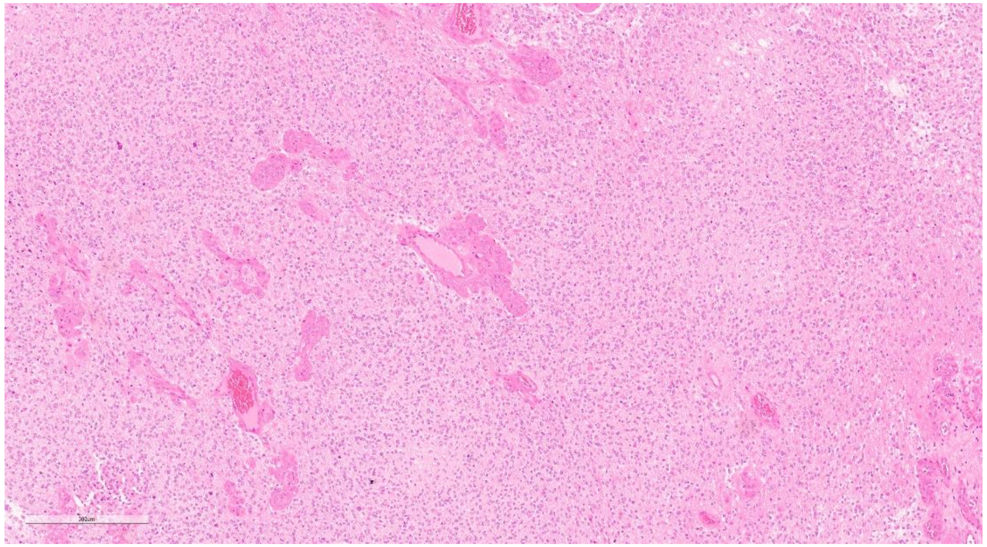

b

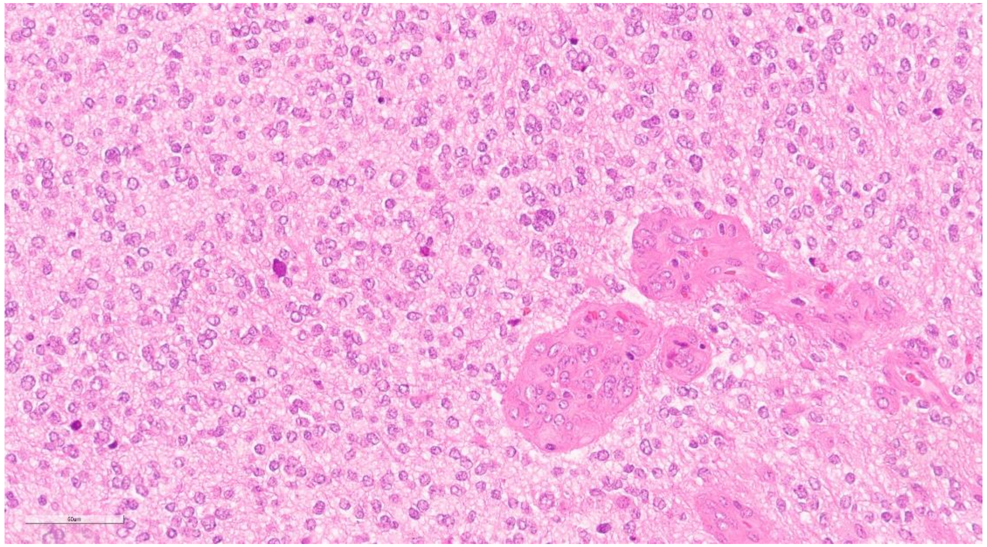

c

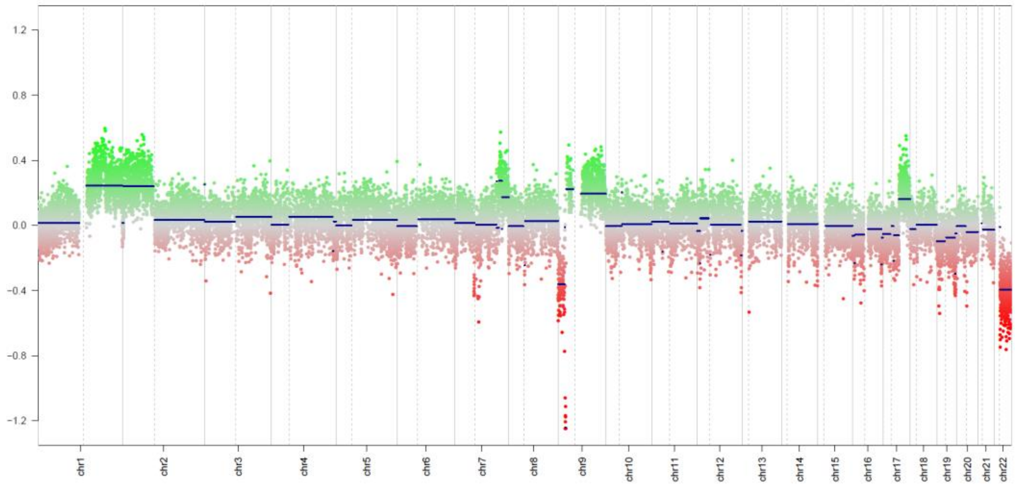

d

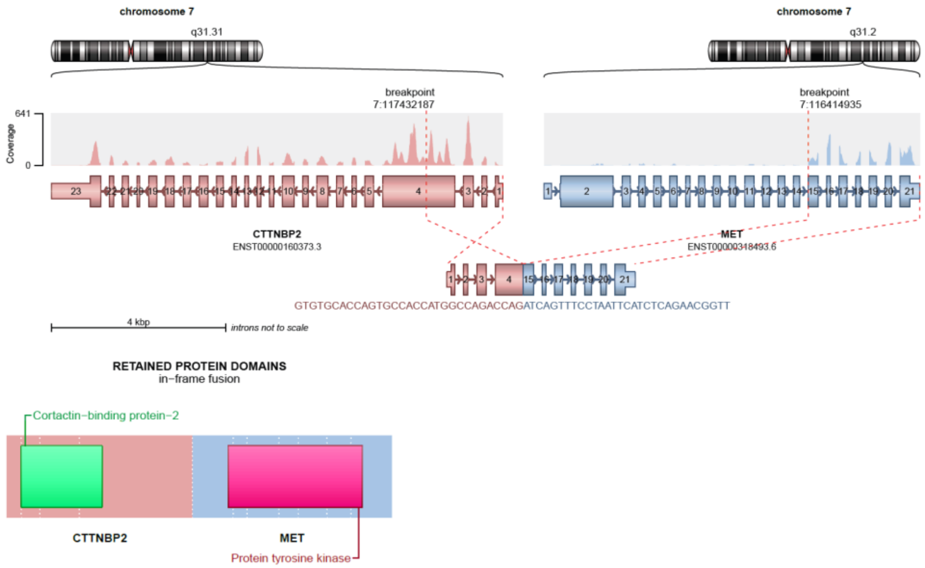

Suppl. Fig. 17: Representative H&E pictures (a, b), the copy-number profile derived from DNA methylation data (c) and a visualization of the gene fusion detected by RNA sequencing (d) of case #16 are presented.

a

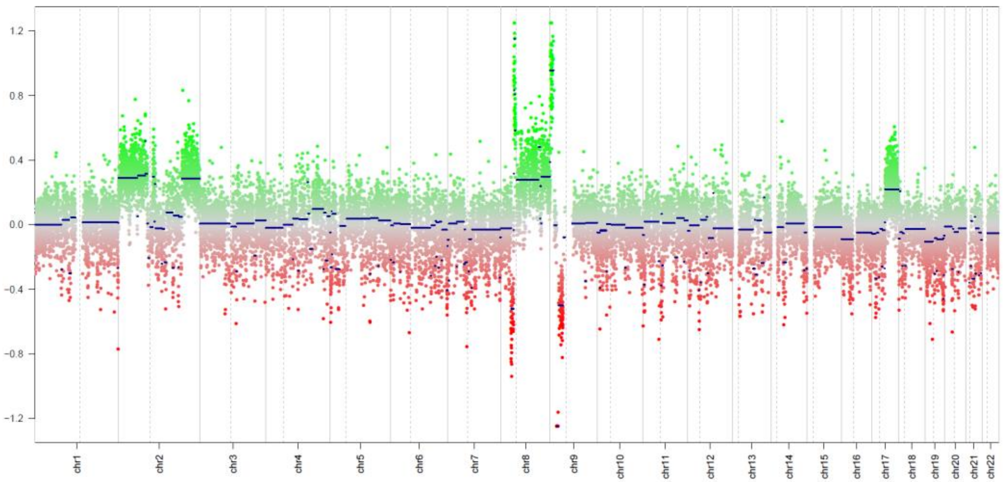

b

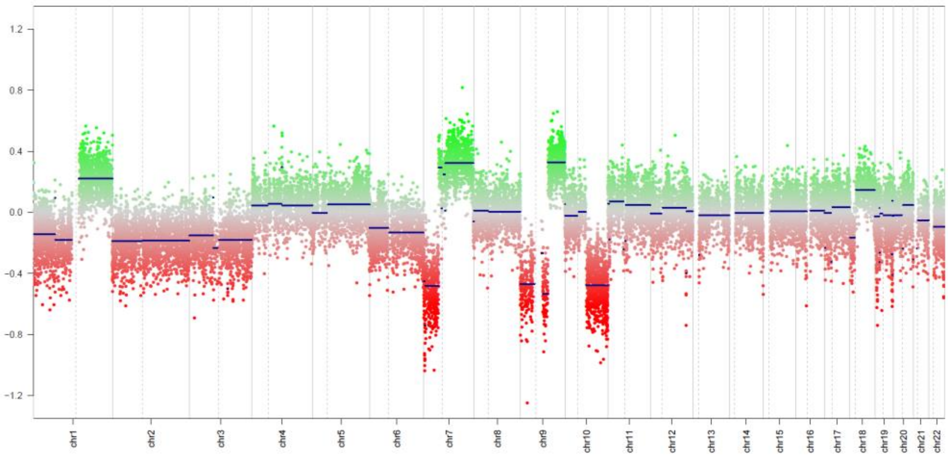

c

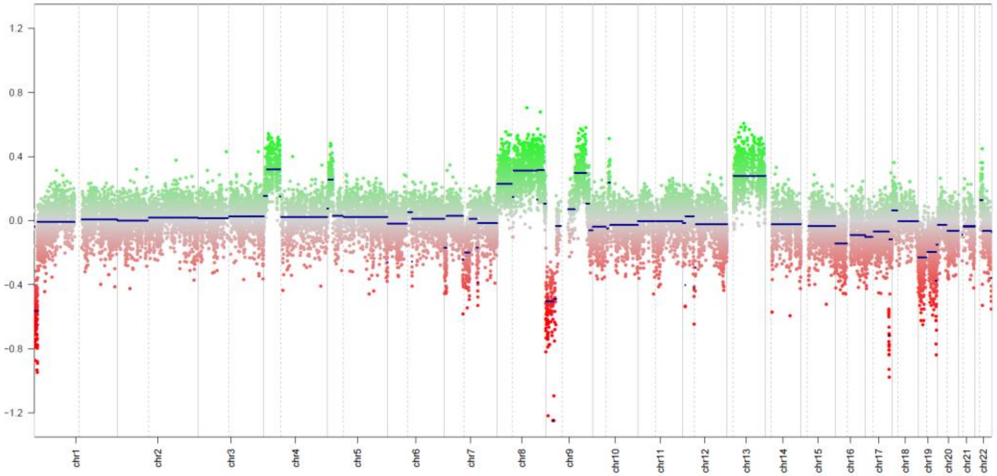

d

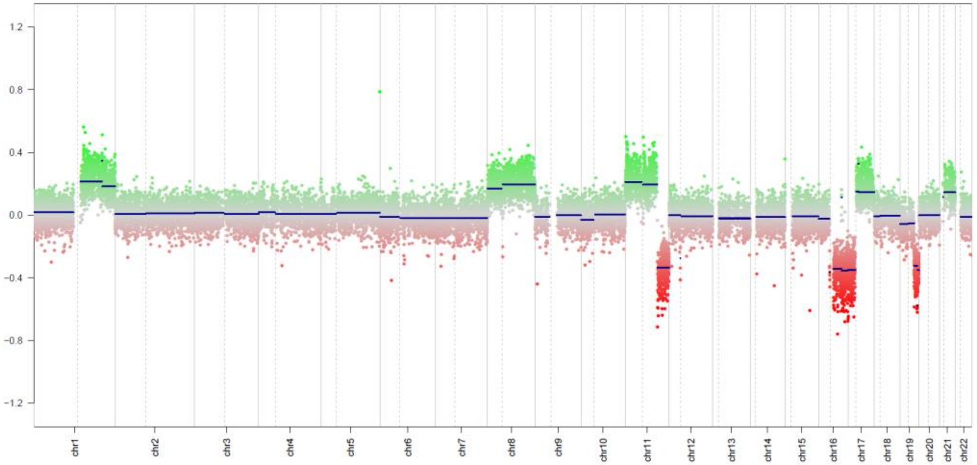

Suppl. Fig. 18: Copy-number profiles derived from DNA methylation data for cases #17 (a), #18 (b), #19 (c) and #20 (d) are presented. No tissue was available for further histological workup and fusion analysis.
